# Role of the HCF-1 Basic Region in Sustaining Cell Proliferation

**DOI:** 10.1371/journal.pone.0009020

**Published:** 2010-02-02

**Authors:** Marco Mangone, Michael P. Myers, Winship Herr

**Affiliations:** 1 Watson School of Biological Sciences, Cold Spring Harbor Laboratory, Cold Spring Harbor, New York, United States of America; Agency for Science, Technology and Research (A*STAR), Singapore

## Abstract

**Background:**

The human herpes simplex virus-associated host cell factor 1 (HCF-1) is a conserved human transcriptional co-regulator that links positive and negative histone modifying activities with sequence-specific DNA-binding transcription factors. It is synthesized as a 2035 amino acid precursor that is cleaved to generate an amino- (HCF-1_N_) terminal subunit, which promotes G1-to-S phase progression, and a carboxy- (HCF-1_C_) terminal subunit, which controls multiple aspects of cell division during M phase. The HCF-1_N_ subunit contains a Kelch domain that tethers HCF-1 to sequence-specific DNA-binding transcription factors, and a poorly characterized so called “Basic” region (owing to a high ratio of basic vs. acidic amino acids) that is required for cell proliferation and has been shown to associate with the Sin3 histone deacetylase (HDAC) component. Here we studied the role of the Basic region in cell proliferation and G1-to-S phase transition assays.

**Methodology/Principal Findings:**

Surprisingly, much like the transcriptional activation domains of sequence-specific DNA-binding transcription factors, there is no unique sequence within the Basic region required for promoting cell proliferation or G1-to-S phase transition. Indeed, the ability to promote these activities is size dependent such that the shorter the Basic region segment the less activity observed. We find, however, that the Basic region requirements for promoting cell proliferation in a temperature-sensitive tsBN67 cell assay are more stringent than for G1-to-S phase progression in an HCF-1 siRNA-depletion HeLa-cell assay. Thus, either half of the Basic region alone can support G1-to-S phase progression but not cell proliferation effectively in these assays. Nevertheless, the Basic region displays considerable structural plasticity because each half is able to promote cell proliferation when duplicated in tandem. Consistent with a potential role in promoting cell-cycle progression, the Sin3a HDAC component can associate independently with either half of the Basic region fused to the HCF-1 Kelch domain.

**Conclusions/Significance:**

While conserved, the HCF-1 Basic region displays striking structural flexibility for controlling cell proliferation.

## Introduction

The herpes simplex virus (HSV) host-cell factor HCF-1 is a key regulator of multiple steps in the human cell-division cycle. Human HCF-1 is a heterodimeric complex of noncovalently associated amino- (HCF-1_N_) and carboxy- (HCF-1_C_) terminal subunits derived by proteolytic maturation of a 2035-amino-acid precursor protein [1]–[3]. The HCF-1_N_ subunit is required for progression through the G1 phase of the cell cycle — depletion of the HCF-1_N_ subunit leads to a G1 phase arrest — and the HCF-1_C_ subunit is required for proper M-phase progression — depletion of the HCF-1_C_ subunit leads to defects in mitotic histone modification, chromosome segregation, and cytokinesis leading to multinucleated cells [4]–[8]. Here, we have studied the structure and function of the HCF-1_N_ subunit in cell proliferation and G1-to-S phase progression.

The HCF-1_N_ subunit consists of three identified functional regions: an amino-terminal Kelch domain, a short self-association sequence (SAS1) involved in noncovalent association with the HCF-1_C_ subunit, and a so-called Basic region (residues 478 to 875) because whereas it is composed of 8% lysine and arginine residues (30 total) it has only one acidic residue (reviewed in [9]). Of these three regions, the Kelch domain and SAS1 elements are the best characterized. The Kelch domain contains six Kelch repeats and is predicted to form a β propeller structure [10]. β propellers are a structural motif commonly involved in protein-protein association. In HCF-1, it is responsible for the ability of HCF-1 to bind to the HSV virion protein VP16 and stabilize a transcriptional regulatory complex with the cellular transcription factor Oct-1, which activates HSV immediate-early transcription during lytic infection (reviewed in [9]). The Kelch domain also tethers HCF-1 to chromatin in uninfected cells through association with sequence-specific DNA-binding proteins (reviewed in [9]). In contrast, the SAS1 element consists of a short 43-amino-acid region of no known structure that binds to two Fibronectin type III repeats in the HCF-1_C_ subunit [11].

The nature of the Kelch domain and SAS1 sequence has been characterized by multiple mutational studies involving careful deletion or amino-acid substitution analysis [10]–[13]. In contrast, the nature of the Basic region has been less extensively characterized: A deletion analysis has shown that it is required for the ability of the HCF-1_N_ subunit to support G1-phase progression [11] whereas protein–protein interaction studies have shown that portions of the Basic region interact with the sequence-specific transcription factors Sp1 [15] and GABP [16], and the Sin3 histone deacetylase (HDAC) [17].

Here, we have performed an extensive mutational analysis of the HCF-1 Basic region and characterized its role in G1-phase progression and association with the Sin3 HDAC. Our results indicate that the Basic region contains multiple elements that can contribute to G1-phase progression provided they are present in sufficient numbers.

## Methods

### Preparation of HCF-1 Basic Region Mutants

The pBABE XBC retroviral parent vector has been described elsewhere [5]. HCF-1 Basic region mutants carrying the HCF-1 siRNA-resistant mutation described previously [6] were prepared using the Quick Change system according to the manufacturer protocol (Stratagene). See Table S1 for the primer list. The HCF-1 duplication mutants have been prepared targeting the HCF-1 Basic region sequence from amino acid 450–725 (D1) and from amino acid 726–1000 (D2). PCR amplicons of these regions were used to prepare the tandem clones with site directed mutagenesis used to make a perfect in frame fusion of the duplicated half junction.

Each HCF-1 Basic region deletion mutant, including HCF-1_N1011Δ381–1000_ and HCF-1_N1011Δ451–1000_, contains carboxy-terminal amino acids starting from amino acid 1001 to amino acid 1011. Following the preparations, all the clones have been sequenced within the deletion borders and tested in COS7 or 293 cells for faithful expression. All HCF-1 mutants have been engineered to contain a FLAG tag and an HA tag at 5′ of the transcription start site.

### tsBN67 Proliferation Assay

tsBN67 cells were plated at a concentration of 1×10^5^ per 10 cm petri dish and kept overnight at 33.5°C in Dulbecco's modified Eagle's medium (DMEM) media containing 10% fetal bovine serum (FBS). The next day, cells were transfected with 2 µg of the appropriate construct using Fugene 6 reagent according the manufacturer's instructions (Roche) and kept at 33.5°C overnight. The next day, cells were trypsinized, and resuspended in 1 ml of DMEM 10% FBS. 1/10 of the cells (∼100,000 cells) were plated at the nonpermissive temperature (40°C) for 14 days with puromycin selection (2 µg/ml) and 1/20 of the cells (∼50,000 cells) were plated at permissive temperature (33.5°C) for 10 days also with puromycin selection (4 µg/ml; the higher puromycin concentration is owing to the higher antibiotic resistance of the cells at the lower temperature). Each clone was tested in at least three rounds of independent transfections plated in duplicate. At the conclusion of the experiment, cells were washed twice in PBS, fixed with 3% formaldehyde for 20 minutes and stained with crystal violet overnight. The next day, plates were washed in deionized H_2_O to remove the excess stain, dried at room temperature, and photographed. For the spectrophotometric assay, the stain was recovered with 1 ml of 2% acetic acid solution and the absorbance measured at 595 nm. The data were normalized by determining the OD_595_ ratio of the permissive versus nonpermissive temperature samples and expressed as a percentage of HCF-1_N1011_.

### Preparation of Stable HeLa Cell Lines

To prepare retrovirus vector stocks, Phoenix ampho cells were plated at the concentration of 5×10^5^ in 10 ml DMEM, 10% FBS, 1% penicillin-streptomycin, 1% glutamine per 10 cm dish and grown at 37°C overnight. The cells were transfected with Fugene 6 reagent containing 2 µg of vector DNA as described above. Cells were grown for 12 hours at 37°C. The following day the supernatant from the Phoenix ampho cells was collected, filtered through a 0.45 µm filter and 3 µl polybrene (5 mg/ml) was added to each sample. The supernatants were used to infect HeLa cells plated at a concentration of 5×10^5^ cells per 10 cm plate in DMEM, 10% FBS, 1% penicillin-streptomycin, 1% glutamine. To promote infection, the plated HeLa cells with virus were centrifuged for 10 min at 1000 rpm at room temperature and subsequently incubated at 37°C. For each construct, we performed three rounds of infections repeated every 6 hours. At the end of the third infection, the HeLa cells were incubated 24 hours in DMEM 10% FBS 1% penicillin streptomycin, 1% glutamine and then exposed to the selection marker (puromycin, 2 µg/ml). Cells were subjected to puromycin selection for three weeks. Twelve individual colonies for each mutant were randomly picked and expanded. Expanded clones were tested for transgene expression by immunoblot using either HA (12CA5) or FLAG (M2) antibodies. The Li-Cor Odyssey system was used for band detection according the manufacturer instructions (Li-Cor).

### HCF-1 siRNA Treatment and BrdU Incorporation Assay

HCF-1 silencing depletion by siRNA treatment, HCF-1 (Rhodamine) and BrdU (FITC) staining for determination of endogenous HCF-1 depletion and cell-cycle progression analysis, respectively, and fluorescent-cell picture acquisition were as described [6]. Six cover slips per mutant cell line were used: two each for cells treated with the HCF-1 siRNA, Luciferase siRNA, and mock treated. Ten pictures for each cover slip were taken using the modular imaging Openlab software (www.improvision.com). Each picture was then processed with the image-processing program ImageJ (http://rsb.info.nih.gov/ij/). Each experiment was repeated three times and 700 to 1900 cells for each cell line were counted.

### Co-Immunoprecipitation Experiments

HeLa-cell extracts were prepared using 300 mM NaCl as previously described [17] with confluent 15 cm culture dishes and immunoprecipitated using 100 µl of FLAG beads (Sigma) for 45 minutes in 100 mM salt. The beads were then washed five times in lysis buffer with 100 mM salt, diluted in SDS Laemmli sample buffer and boiled for 10 minutes. The immunoprecipitates were then resolved by 8% SDS-PAGE and immunoblotted using FLAG (Sigma) or Sin3a (Santa Cruz) antibodies. The Odyssey system was used for band detection according the manufacturer instructions (Li-Cor).

## Results

**Figure 1:**
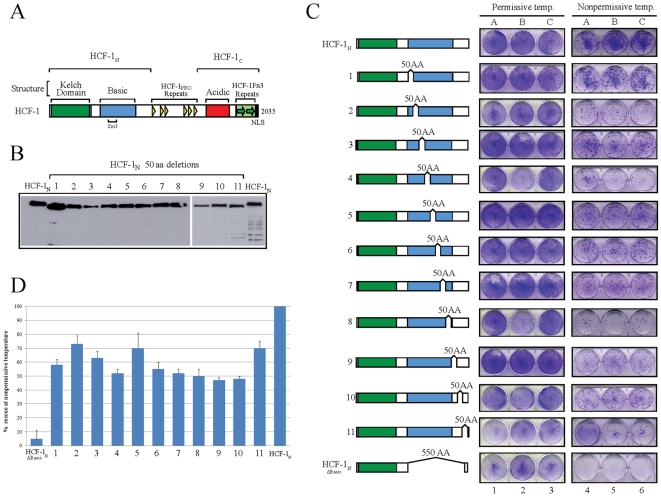
The HCF-1 Basic region does not contain a unique essential region to promote cell proliferation. A) Schematic structure of human HCF-1. Structural elements and subunit boundaries are shown above. The small bracket below the figure highlights the Sin3 binding site. B) Western blot analysis of the expression of the eleven 50 AA deletion-scanning mutants (labeled 1 to 11) in COS7 cells. C) Schematic of the eleven 50 AA scanning deletion mutants (labeled 1 to 11) and tsBN67 colony assay. Plates resulting from growth at the control permissive (left) and selective nonpermmissive (right) temperatures are shown. Each clone has been tested in triplicate as shown in columns A, B, and C. D) Spectrophotometric quantitation of the ability of deletion mutants to rescue cell proliferation at the nonpermissive temperature. The error bars are standard error of the mean.

Figure 1A shows a diagram of human HCF-1 illustrating its structural features (see Fig. 1A legend and [9] for details). The 2035 aa HCF-1 precursor undergoes proteolytic maturation through successive cleavages at any one of six central sequence repeats called HCF-1_PRO_ repeats to generate the HCF-1_N_ and HCF-1_C_ subunits. As illustrated in Figure 1A, the HCF-1_N_ subunit has two important segments: the Kelch domain and Basic region. HCF-1 was initially shown to be involved in cell-cycle progression through the study of a temperature-sensitive baby hamster kidney cell line called tsBN67 in which HCF-1 contains a proline-to-serine missense mutation (P134S) in the HCF-1 Kelch domain [4], [18]. These cells grow at the permissive temperature of 33.5°C but stop proliferating at 40°C and enter a stable arrest. Growth at 40°C can be rescued, however, by transfection of the wild-type HCF-1 gene in a colony formation assay [4]. It is using this colony-formation assay that Wilson et al. [10] showed that the HCF-1_N_ subunit is sufficient and that the HCF-1_N_ Kelch domain and Basic region are each required to rescue tsBN67-cell growth at 40°C. We used the same assay to determine the sequences within the HCF-1_N_ Basic region required to support tsBN67 cell proliferation at 40°C.

We initially prepared and tested a set of eleven 50 amino acid scanning deletion mutant constructs prepared using the HCF-1_N1011_ subunit as template and beginning with residue 451 and ending with residue 1000 (labeled 1–11 in Fig. 1C). Each of these mutant constructs was initially tested for faithful mutant protein synthesis after transient expression in 293 cells as shown in Figure 1B. Although there was some variation in the levels of synthesis, all of the 50 amino acid deletion mutants migrated somewhat faster than the parental HCF-1_N_ subunit construct during PAGE, indicating successful in-frame deletion of each 50 amino acid segment. The mutants were then assayed for their ability to rescue tsBN67 cell proliferation at the 40°C nonpermissive temperature.

### No Unique Region within the HCF-1 Basic Region Is Required to Maintain Cell Proliferation

The results of such an experiment are shown in the form of a colony formation assay in Figure 1C and quantitated in Figure 1D. In the experiment shown, tsBN67 cells were transfected with each HCF-1 construct in triplicate and grown under antibiotic selection at either the permissive temperature of 33.5°C or nonpermissive temperature of 40°C. After 10 and 14 days at 33.5°C and 40°C, respectively, the plates were stained with crystal violet to identify colonies (Fig. 1C, enlarged pictures are shown in Fig. S1), and the crystal violet stain extracted and quantitated spectrophotometrically (Fig. 1D). As expected, the HCF-1_N_-subunit construct but not the HCF-1 _NΔBasic_ construct lacking the entire Basic region (lacking residues 451–1000) rescued tsBN67-cell proliferation (Fig. 1C and D). To our surprise, albeit to somewhat reduced levels, all of the 11 50 aa scanning deletion mutants tested were able to rescue tsBN67 cell proliferation at the nonpermissive temperature. Although the level of rescue by each mutant is not necessarily identical, in contrast to the HCF-1 _NΔBasic_ construct, all of the 50 amino acid deletion mutants supported colony formation (Fig. 1C). These results suggest that there is no individual sequence element within the Basic region that is essential for HCF-1_N_-subunit rescue of the tsBN67 cell-proliferation defect.

### N- and C-Terminal HCF-1 Basic Region Deletions of Increasing Size Result in Progressive Loss of tsBN67 Cell-Proliferation Rescue

**Figure 2:**
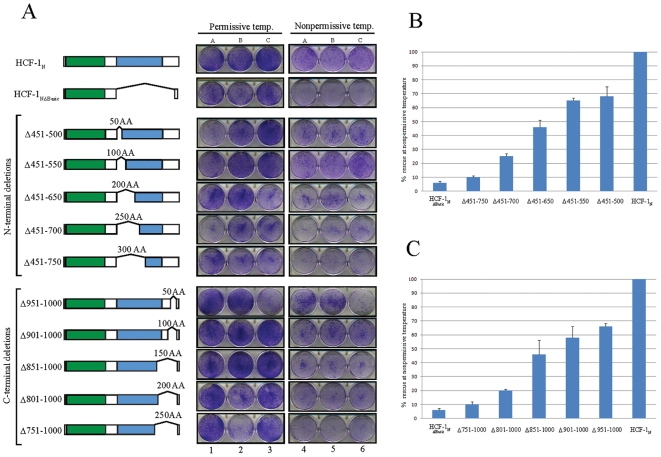
HCF-1 Basic region deletion mutants rescue cell proliferation in a size-dependent manner. A) Schematics of the nested set of N-terminal and C-terminal HCF-1 deletion mutants and tsBN67 colony assay. B and C) Spectrophotometric quantitation of the ability of N- and C-terminal deletion mutants to rescue cell proliferation at the nonpermissive temperature. The error bars are standard error of the mean.

The failure to map a discrete region within the HCF-1 Basic region necessary to rescue tsBN67-cell proliferation at nonpermissive temperature could be due to the activity of redundant elements within the HCF-1 Basic region. To test this hypothesis, we created and assayed a series of N- and C-terminal HCF-1 Basic region deletions of increasing size as shown in Figure 2. Each of these mutant constructs was faithfully expressed in the 293-cell transient expression assay (see Fig. S2). Although all of these deletion constructs were able to support colony formation at nonpermissive temperature albeit to differing degrees (Fig. 2A; enlarged pictures are shown in Fig. S2), there was a marked diminishment in both the size and number of colonies as clearly evidenced by the spectrophotometric assay (Fig. 2B and C). Consistent with the scanning deletion analysis described above, these results suggest that there is no unique essential element required to sustain tsBN67-cell proliferation but that there is a size dependency in Basic region function. Thus, the HCF-1 Basic region seems to possess an intrinsic plasticity, with multiple elements able to rescue cell proliferation.

### Duplications of N- and C-Terminal HCF-1 Basic Region Segments Restore tsBN67-Cell Proliferation Rescue

**Figure 3:**
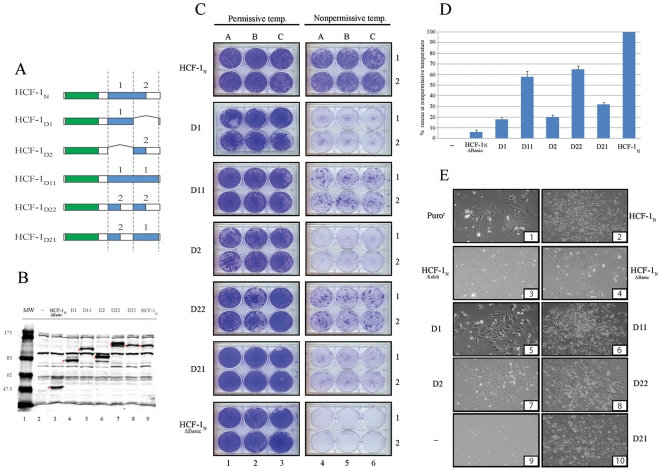
HCF-1 Basic region duplications can rescue tsBN67 colony forming activity at the nonpermissive temperature. A) Schematic of the HCF-1 Basic region duplication mutants. Each domain, labeled 1 and 2, has been selectively deleted, duplicated or placed in inverted position. B) Western blot analysis of the expression of HCF-1 Basic region mutants in 293 cells. Red asterisks indicate the expected proteins based on their predicted size. C) tsBN67 colony formation assays. Each clone has been tested at permissive and nonpermissive temperature in triplicate as shown in columns A, B, and C and plated in duplicate. D) Spectrophotometric quantitation of the ability of deletion mutants to rescue cell proliferation at the nonpermissive temperature. The error bars are standard error of the mean. E) Colony morphology of selected clones at nonpermissive temperature.

If the HCF-1 Basic region is indeed plastic owing to multiple redundant elements spread throughout the region that act in a cumulative manner, we hypothesized that residual activity in the N- or C-terminal halves of the Basic region (Fig. 2) might be amplified by tandem duplication. We tested this hypothesis by preparing mutants containing such tandem duplications. We divided the HCF-1 Basic region analyzed in these studies (amino acids 451 to 1000) into two equally sized 275 amino acid segments: an N-terminal half called Domain 1 (D1) present in HCF-1_Δ726–1000_ and a C-terminal half called Domain 2 (D2) present in HCF-1_Δ451–725_. We created a set of five HCF-1_N_ mutants, called HCF-1_N-D1_, HCF-1_N-D2_, HCF-1_N-D11_, HCF-1_N-D22_, and HCF-1_N-D21_. The structure of these five mutants is shown in Figure 3A. The HCF-1_N-D1_, and HCF-1_N-D2_ mutants lack the D2 and D1 segments, respectively. The HCF-1_N-D11_ and HCF-1_N-D22_ mutants contain tandemly duplicated D1 and D2 segments, respectively. And in the HCF-1_N-D21_ mutant, the position of the D1 and D2 segments has been swapped. Each of these mutant constructs resulted in faithful mutant protein expression as shown in Figure 3B.

We first tested the two new deletion mutants (HCF-1_N-D1_ and HCF-1_N-D2_) in the tsBN67-cell proliferation rescue assay. Consistent with the sequential N- and C-terminal deletion mutants (see Fig. 2), both mutants, although lacking any shared Basic region sequences, similarly supported a low level of colony formation at the nonpermissive temperature (Fig. 3 C and D; enlarged pictures of the plates are shown in Fig. S3). We next tested the HCF-1_N-D11_ and HCF-1_N-D22_ mutants containing duplicated domain 1 and 2 segments, respectively. Strikingly, both duplication mutants supported an enhanced level of colony formation at the nonpermissive temperature compared to their parental non-duplicated mutants (Fig. 3 C and D). The increased levels of tsBN67-cell proliferation exemplified by the colony formation is also reflected in the higher cell numbers in individual colonies as illustrated in the photomicrographs shown in Figure 3E. Thus, amplification of either HCF-1 Basic region Domain 1 or Domain 2 enhances HCF-1_N_ subunit support of tsBN67-cell proliferation. These results are consistent with the hypothesis that the HCF-1 Basic region contains multiple redundant elements that can cooperate with one another or themselves.

Although the duplicated Domain 1 and 2 appeared to provide considerable activity, swapping the N- and C-terminal arrangement of Domain 1 and 2 in the HCF-1_N-D21_ mutant led to less robust colony formation (Fig. 3C and D) indicating that there are some constraints to Basic region structure and function. Nevertheless, in toto, these results indicate a considerable flexibility in HCF-1 Basic region structure and function.

### HCF-1 Basic Region Domain 1 and 2 Support G1-to-S Progression upon Depletion of HCF-1 in HeLa Cells

The tsBN67-cell proliferation assay used in the aforementioned experiments assays the role of the HCF-1 Basic region in supporting cell proliferation but does not define the cell-cycle step(s) involved. Previous studies in HeLa cells have suggested that the HCF-1_N_ subunit is required for the passage from the G1 to S phases of the cell cycle [6] through association with the E2F1 cell-cycle transcription factor [14], [19]. We were interested in whether the HCF-1 Basic region is involved in promoting G1-to-S progression and therefore assayed the activity of the deleted, duplicated, and swapped Domain 1 and 2 mutants in the BrdU incorporation assay described by Julien and Herr [6]. For this assay, we prepared stable cell lines expressing each of the mutant HCF-1_N_ constructs described in Figure 3 in an siRNA-resistant [6] and epitope-tagged form. By preparing these HeLa cell lines, we were also able to compare directly the activity of the various mutants in supporting G1-to-S phase progression to HCF-1_N_-subunit effector-protein interactions described previously ([9]; see below). The protein expression is shown in Figure S4.

**Figure 4:**
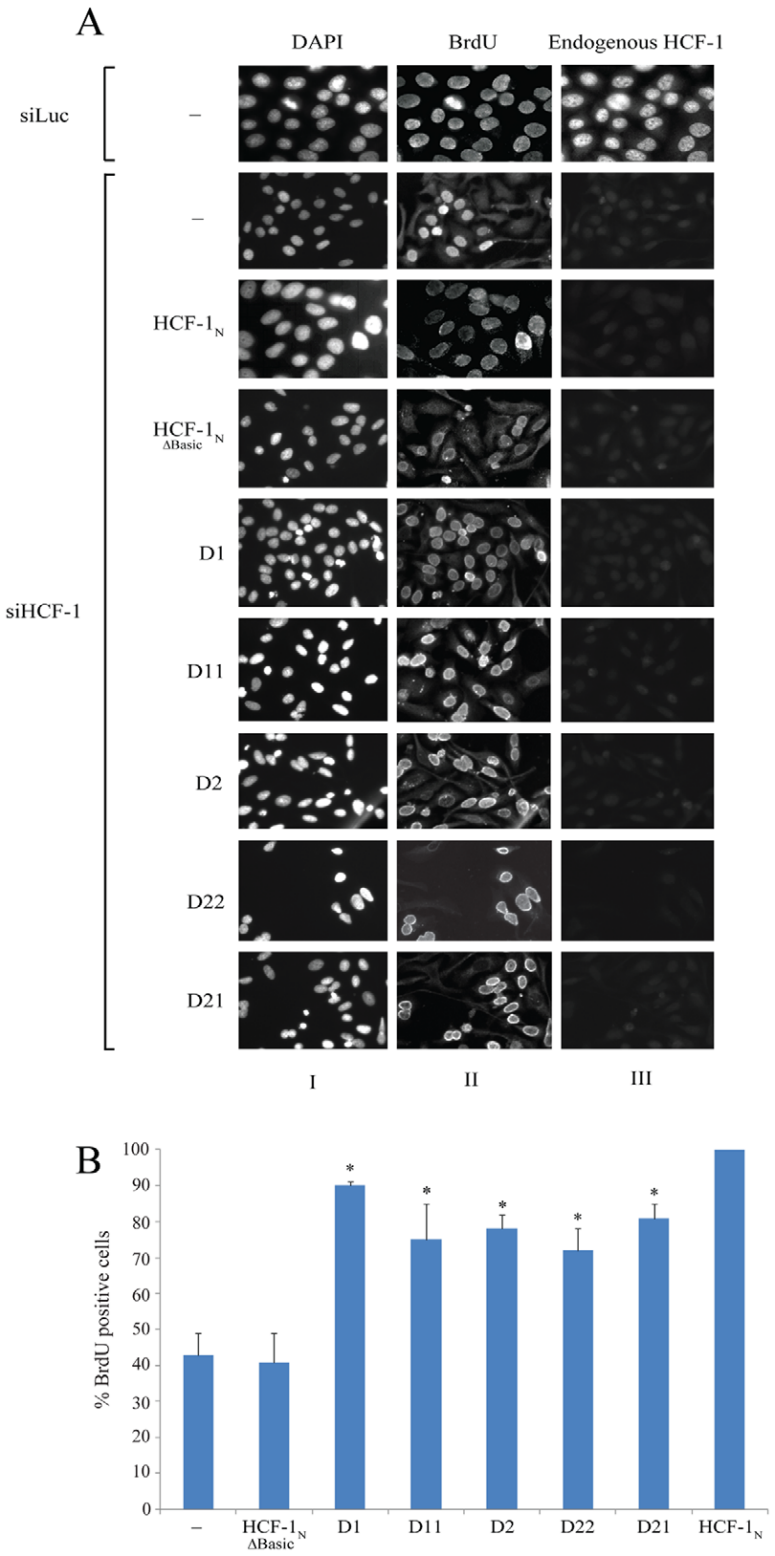
HCF-1 Basic region Domains 1 and 2 can individually promote HeLa cell G1-to-S phase progression. HeLa cells that stably express different HCF-1 silencing resistance mutants were tested for their ability to bypass G1-to-S arrest induced by silencing of endogenous HCF-1 with RNAi. A) Immunofluorescence analysis of HeLa cells silenced with Luciferase RNAi oligos and with HCF-1 siRNA oligos. Three panels are showed for each clone tested. The left panel shows DNA staining with DAPI. The middle panel shows cells probed with antibody for BrdU incorporation. The right panel shows the level of endogenous HCF-1 remaining after siRNA treatment as detected by the HCF-1_C_ antibody (αH12). B) Percentage of BrdU positive cells after 72 hours of siRNA silencing as determined by the immunofluorescence results normalized to HCF-1_N1011_. Sample sizes were as follows: HeLa, n = 1127; HCF-1_N1011-ΔBasic_, n = 881; HCF-1_N1011-D1_, n = 1920; HCF-1_N1011-D11_, n = 859; HCF-1_N1011-D2_, n = 705; HCF-1_N1011-D22_, n = 1798; HCF-1_N1011-D21_, n = 1478; and HCF-1_N1011_, n = 1475. The error bars are standard error of the mean. *p<0.05 (Mann-Whitney U test), comparing the HCF-1_N_ mutants to HCF-1_N1011-ΔBasic_.

For the siRNA G1-to-S phase progression assay, endogenous HCF-1 was depleted by two rounds of siRNA treatment separated by 12 hrs. Forty-eight hours after the first transfection, cells were incubated for a further 24 hrs in the presence of BrdU. In this assay, cells that can enter S phase incorporate BrdU and are identified by anti-BrdU staining. Figure 4 shows the results of this experiment.

Using immunofluorescence analysis, we showed that endogenous HCF-1 was effectively depleted in 90% of cells by the siRNA treatment (Fig. 4A column III and data not shown). As previously described [6], with the parent HeLa cells depleted of endogenous HCF-1, 40% of cells were able to pass from the G1 to S phase and this level was not altered by the Basic region deletion mutant HCF-1 _NΔBasic_ (Fig. 4B). These results indicate that, as for tsBN67-cell proliferation, the Basic region is required for full G1-to-S phase progression in HeLa cells. Interestingly, all of the Basic region Domain 1 and 2 deletion, duplication, and swap mutants supported G1-to-S phase progression at elevated levels between the full HCF-1_N_ subunit and the Basic region deletion mutant HCF-1 _NΔBasic_. This result suggests that the Basic region requirements for G1-to-S phase progression in HeLa cells depleted of HCF-1 by siRNA treatment are less stringent than for tsBN67-cell proliferation rescue as Domain 1 and 2 in single or duplicate copies augment G1-to-S phase progression in the HeLa-cell assay more effectively than tsBN67-cell proliferation.

### Sin3a HDAC Association with HCF-1 Basic Region Mutants Correlates with G1-to-S Phase Function

**Figure 5:**
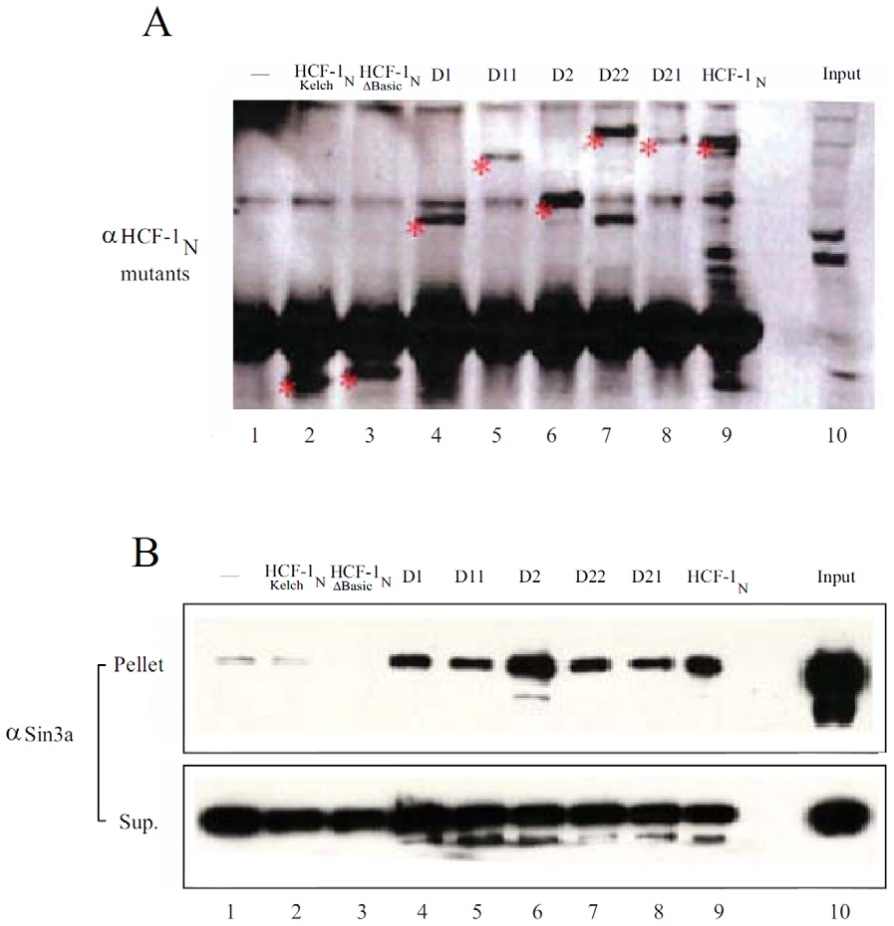
Multiple regions within the HCF-1 Basic region associate with Sin3a. αFLAG immunoprecipitates of the stable HeLa cell lines with the single, duplicated, and swapped Basic region Domains 1 and 2 were analyzed by immunoblot with an αSin3a antisera. A) Recovery of the FLAG tagged HCF-1 recombinant proteins was verified by immunoblot analysis with the αFLAG antibody. B) Sin3a in the supernatant (bottom) and immunoprecipitates (top) was determined by αSin3a immunoblotting.

The aforementioned studies suggest that multiple regions within the HCF-1 Basic region support G1-to-S phase progression. We were surprised by this result because to date there is no common factor that has been shown to interact with both halves of the HCF-1 Basic region that could explain the activity of this region in G1-to-S phase progression. For example, as shown in Figure 1A, the Sin3 HDAC has been shown by yeast two-hybrid analysis to interact with mouse HCF-1 at residues 610–722 in Basic region Domain 1 (these residue numbers are the same in human and mouse HCF-1). The two-hybrid result, however, only reveals a positive interaction and does not exclude other potential interactions between Sin3 and HCF-1. Given that Domains 1 and 2 both have the potential to promote G1-to-S phase progression, we were interested in determining whether there was selectivity in Sin3 association with the two domains. We therefore assayed Sin3 HDAC association with the different Domain 1 and 2 deletion, duplication, and swap HCF-1_N_ mutants as shown in Figure 5.

We performed HCF-1–Sin3a co-immunoprecipitation analyses with the stable HCF-1 cell lines. As shown in Figure 5A, all of the HCF-1 Domain 1 and 2 mutants were faithfully recovered in the anti-FLAG HCF-1 immunoprecipitation. Consistent with the activity of the Domain 1 and 2 deletion, duplication, and swap mutants in supporting G1-to-S phase progression, they were all able to associate with the Sin3a HDAC (Fig. 5B, lanes 4–9), whereas the inactive HCF-1 _NΔBasic_ mutant did not associate with Sin3a (compare lanes 1–3). These results suggest that there are multiple binding sites for Sin3a on the HCF-1 Basic region and that its association with the HCF-1 Basic region correlates with the ability of this region to support G1-to-S phase progression in the HeLa-cell assay. They do not exclude, however, interactions also with the Kelch domain, which is shared with all of the constructs shown in Figure 5.

## Discussion

Here we have analyzed the structure and function of the Basic region of HCF-1. Unexpectedly, while essential for HCF-1 function in cell-cycle progression, it displays a flexible albeit size dependent structure, as all of the sequential 50 amino acid deletions had relatively little effect on the ability of the HCF-1_N_ subunit to support tsBN67 cell proliferation at the nonpermissive temperature. Furthermore, while each half of the Basic region displays little activity on its own, simple duplicates of each half can restore cell-proliferation activity. Such structural and functional properties are reminiscent of transcriptional activation domains many of which have been characterized as enriched in acidic, proline, or glutamine residues [20].

It is not known what character of the Basic region might be important for its activity. It is indeed important to reiterate (see Introduction) that, while referred to as a Basic region, this region is not particularly enriched in basic residues but rather more lacking in acidic residues, and thus it is very possible that it is some other property besides basic residues that is important for the function of the Basic region. Such properties could be enrichment of other amino acids (e.g., the region is enriched in valine and threonine residues) or short motifs, which can also be found (data not shown).

Nevertheless, an importance of the overall basic (non-acidic) nature of the HCF-1 Basic region is underscored by its conservation among vertebrate and many invertebrate HCF-1 molecules (see for example [21]). Indeed, even though its structure seems very malleable, the amino acid sequence of vertebrate HCF-1 molecules displays considerable sequence conservation. For example, the region from amino acid 513 to 781 of human HCF-1 is 83% identical in Zebrafish HCF-1. In contrast, insect HCFs display conserved basic (non-acidic) character without a human-sequence identity, emphasizing an unusual importance of a basic (non-acidic) character for Basic region function. Nevertheless, Basic regions are not universally conserved in HCF molecules as HCF-1 in the worm *C. elegans* displays little basic character.

These differences in Basic region conservation may indeed reflect functional differences. Recent studies have revealed that human and Drosophila HCF molecules interact with both activating (E2F1) and repressive (E2F4 in humans and E2F2 in Drosophila) members of the E2F family of cell-cycle transcriptional regulators [19]. These interactions are promoted by the binding of the HCF-1 Kelch domain with the so called HCF-1 binding motif (HBM) loosely defined by the sequence ^E^/_D_HxY where x denotes any amino acid and this association is important for promoting cell-cycle progression in human cells ([19], [23]). Interestingly, *C. elegans* HCF-1 has neither a well-defined Basic region nor do *C. elegans* E2F molecules possess HBMs. Thus the role of HCF-1 in cell-cycle progression may vary in the worm.

We have used two assays to study the role of the HCF-1 Basic region in mammalian cell-cycle progression: rescue of tsBN67 cell proliferation at nonpermissive temperature and G1-to-S progression in HeLa cells depleted of HCF-1 by siRNA treatment. These two assays identify different requirements for the Basic region. Thus, the tsBN67 rescue assay is more stringent than the HeLa-cell G1-to-S progression assay because single-copy Domain 1 and 2 constructs are relatively less active in the tsBN67 rescue assay than the HeLa-cell G1-to-S phase progression assay. These differences may well reflect differences in how HCF-1 is inactivated in the two assays (i.e., elevated temperature vs. siRNA depletion) or different genetic backgrounds of these two cell lines. In the first case, it is possible that residual endogenous HeLa-cell HCF-1 remaining after siRNA treatment is sufficient to support some but not all aspects of G1-to-S progression and that only Domain 1 or 2 alone is required to provide the missing activity. In the different genetic background case, the presence in HeLa cells of the human papilloma virus E7 gene product, which inhibits retinoblastoma protein (pRb) function, may make these cells less stringent for HCF-1 Basic region-mediated G1-to-S phase progression. We have previously shown that loss of pRb function in tsBN67 cells can rescue the temperature-sensitive defect [22]. Thus it is reasonable to hypothesize that the requirements for HCF-1 to promote cell-cycle progression are more relaxed in HeLa cells. Furthermore, the G1-to-S progression assay is likely to be more limited in scope than a rescue of complete cell proliferation in the tsBN67 cell assay.

We note with interest, however, that Sin3a association with the HCF-1 Basic region mutants in HeLa cells correlates with the ability of these mutants to support G1-to-S phase progression in these same cells. This correlation suggests that Sin3 association with HCF-1 is important in promoting the G1-to-S phase transition. At present we do not know what precise role this interaction might play as for example in the regulation of transcription of a specific set of genes.

 HCF-1 interacts with both the MLL family of histone H3 K4 methyltransferases (via the Kelch domain) and Sin3 (via the Basic region) and can do so simultaneously even though they are associated with opposite transcriptional outcomes, activation for the methyltransferases and repression for the histone deacetylase [17]. Interestingly, however, when HCF-1 is associated with the repressive E2F4 molecule, the complex is preferentially associated with Sin3 whereas, when HCF-1 is associated with the activator E2F1 molecule to promote G1-to-S phase progression [23], HCF-1 is preferentially associated with H3K4 methyltransferases [19]. Thus it is not yet readily evident how HCF-1 Basic region association with Sin3 histone deacetylase activity might translate into sustaining G1-to-S phase progression. Perhaps there is a two step cell-cycle regulatory process whereby first the Basic region plays an essential role by repressing transcription and then the Kelch domain plays an essential role by activating transcription. Such a two-step regulatory process could be important in assuring that DNA replication is not activated before the G1-to-S phase transition, which can lead to DNA damage and associated detrimental effects such as tumor progression or apoptosis.

## Supporting Information

Table S1 List of the primers used in this manuscript.
(0.09 MB DOC)

Figure S1 tsBN67 colony assay with the 11 scanning deletion mutants.
(2.40 MB PDF)

Figure S2 tsBN67 colony assay with the N-and C-terminal deletion mutants.
(2.12 MB PDF)

Figure S3 tsBN67 colony assay with the duplication and deletion mutants.
(1.86 MB PDF)

Figure S4 Stable Expression of selected HCF-1 N-terminal deletion mutants in HeLa cells.
(0.36 MB PDF)

## References

[1] Wilson AC, LaMarco K, Peterson MG, Herr W (1993). The VP16 accessory protein HCF is a family of polypeptides processed from a large precursor protein.. Cell.

[2] Wilson AC, Peterson MG, Herr W (1995). The HCF repeat is an unusual proteolytic cleavage signal.. Genes Dev.

[3] Kristie TM, Pomerantz JL, Twomey TC, Parent SA, Sharp PA (1995). The cellular C1 factor of the herpes simplex virus enhancer complex is a family of polypeptides.. J Biol Chem.

[4] Goto H, Motomura S, Wilson AC, Freiman RN, Nakabeppu Y (1997). A single-point mutation in HCF causes temperature-sensitive cell-cycle arrest and disrupts VP16 function.. Genes Dev.

[5] Reilly PT, Herr W (2002). Spontaneous reversion of tsBN67 cell proliferation and cytokinesis defects in the absence of HCF-1 function.. Exp Cell Res.

[6] Julien E, Herr W (2003). Proteolytic processing is necessary to separate and ensure proper cell growth and cytokinesis functions of HCF-1.. EMBO J.

[7] Julien E, Herr W (2004). A switch in mitotic histone H4 lysine 20 methylation status is linked to M phase defects upon loss of HCF-1.. Mol Cell.

[8] Lee S, Horn V, Julien E, Liu Y, Wysocka J (2007). Epigenetic regulation of histone H3 serine 10 phosphorylation status by HCF-1 proteins in C. elegans and mammalian cells.. PLoS ONE.

[9] Wysocka J, Herr W (2003). The herpes simplex virus VP16-induced complex: the makings of a regulatory switch.. Trends Biochem Sci.

[10] Wilson AC, Freiman RN, Goto H, Nishimoto T, Herr W (1997). VP16 targets an amino-terminal domain of HCF involved in cell cycle progression.. Mol Cell Biol.

[11] Wilson AC, Boutros M, Johnson KM, Herr W (2000). HCF-1 amino- and carboxy-terminal subunit association through two separate sets of interaction modules: involvement of fibronectin type 3 repeats.. Mol Cell Biol.

[12] Freiman RN, Herr W (1997). Viral mimicry: common mode of association with HCF by VP16 and the cellular protein LZIP.. Genes Dev.

[13] Mahajan SS, Wilson AC (2000). Mutations in host cell factor 1 separate its role in cell proliferation from recruitment of VP16 and LZIP.. Mol Cell Biol.

[14] Knez J, Piluso D, Bilan P, Capone JP (2006). Host cell factor-1 and E2F4 interact via multiple determinants in each protein.. Mol Cell Biochem.

[15] Gunther M, Laithier M, Brison O (2000). A set of proteins interacting with transcription factor Sp1 identified in a two-hybrid screening.. Mol Cell Biochem.

[16] Vogel JL, Kristie TM (2000). The novel coactivator C1 (HCF) coordinates multiprotein enhancer formation and mediates transcription activation by GABP.. EMBO J.

[17] Wysocka J, Myers MP, Laherty CD, Eisenman RN, Herr W (2003). Human Sin3 deacetylase and trithorax-related Set1/Ash2 histone H3-K4 methyltransferase are tethered together selectively by the cell-proliferation factor HCF-1.. Genes Dev.

[18] Nishimoto T, Takahashi T, Basilico C (1980). A temperature-sensitive mutation affecting S-phase progression can lead to accumulation of cells with a G2 DNA content.. Somatic Cell Genet.

[19] Tyagi S, Chabes AL, Wysocka J, Herr W (2007). E2F activation of S phase promoters via association with HCF-1 and the MLL family of histone H3K4 methyltransferases.. Mol Cell.

[20] Mitchell PJ, Tjian R (1989). Transcriptional regulation in mammalian cells by sequence-specific DNA binding proteins.. Science.

[21] Capotosti F, Hsieh JJ, Herr W (2007). Species selectivity of mixed-lineage leukemia/trithorax and HCF proteolytic maturation pathways.. Mol Cell Biol.

[22] Reilly PT, Wysocka J, Herr W (2002). Inactivation of the retinoblastoma protein family can bypass the HCF-1 defect in tsBN67 cell proliferation and cytokinesis.. Mol Cell Biol.

[23] Tyagi S, Herr W (2009). E2F1 mediates DNA damage and apoptosis through HCF-1 and the MLL family of histone methyltransferases.. EMBO J. in press.

