# Supplementary material for: Role of the HCF-1 Basic Region in Sustaining Cell Proliferation
Source: PLoS One. 2010 Feb 2;5(2):e9020. doi: 10.1371/journal.pone.0009020 (PMC2814863; doi:10.1371/journal.pone.0009020)
Supplement: Table S1 — List of the primers used in this manuscript. (0.09 MB DOC) [file pone.0009020.s001.doc]

**Table S1: Primer list.**

1. 50 amino acid scanning deletion mutants:

| 1. 5’HCF-1_N1011Δ541-500_ | TAGTGCGAGGACGGGAACCAGTGGTACGCT |
| --- | --- |
| 1. 3’HCF-1_N1011Δ541-500_ | AGCGTACCACTGGTTCCCGTCCTCGCACTA |
| 1. 5’HCF-1 _N1011Δ501-550_ | TGTTGTCCTTGAGGTCGGCGACGCCGGCGA |
| 1. 3’HCF-1 _N1011Δ501-550_ | TCGCCGGCGTCGCCGACCTCAAGGACAACA |
| 1. 5’HCF-1 _N1011Δ551-600_ | CCCTACCGGCGTGACCAGTACCACTCGTTG |
| 1. 3’HCF-1 _N1011Δ551-600_ | CAACGAGTGGTACTGGTCACGCCGGTAGGG |
| 1. 5’HCF-1 _N1011Δ601-650_ | CACGGAGGAGCGGTCACTGGTGTCAACAC |
| 1. 3’HCF-1 _N1011Δ601-650_ | GTGTTGACACCAGTGACCGCTCCTCCGTG |
| 1. 5’HCF-1 _N1011Δ651-700_ | GTCGTTCGGGTCCACGTCCGCAGGTGCCCA |
| 1. 3’HCF-1 _N1011Δ651-700_ | TGGGCACCTGCGGACGTGGACCCGAACGAC |
| 1. 5’HCF-1 _N1011Δ701-1000_ | AGTCGTCAGTGTCCGTAGGACCCGTAGTCG |
| 1. 3’HCF-1 _N1011Δ701-1000_ | CGACTACGGGTCCTACGGACACTGACGACT |
| 1. 5’HCF-1 _N1011Δ751-800_ | CCCTGGTTCGGGTGGTGGTGGTTCCACTAC |
| 1. 3’HCF-1 _N1011Δ751-800_ | GTAGTGGAACCACCACCACCCGAACCAGGG |
| 1. 5’HCF-1 _N1011Δ801-850_ | GGGTAGTGGTAGTAGGGGTACCCCCCACAA |
| 1. 3’HCF-1 _N1011Δ801-850_ | TTGTGGGGGGTACCCCTACTACCACTACCC |
| 1. 5’HCF-1 _N1011Δ851-900_ | TAGGAGGCGTGACACGTGTCGTGATCACGA |
| 1. 3’HCF-1 _N1011Δ851-900_ | TCGTGATCACGACACGTGTCACGCCTCCTA |
| 1. 5’HCF-1 _N1011Δ901-950_ | CGGCCCCGCCCCCCGTGGTACGTCGGGCAC |
| 1. 3’HCF-1 _N1011Δ901-950_ | GTGCCCGACGTACCACGGGGGGCGGGGCCG |
| 1. 5’HCF-1 _N1011Δ951-1000_ | TGTTGGGGTTGGTAGCCGGTCCCACTACAC |
| 1. 3’HCF-1 _N1011Δ951-1000_ | GTGTAGTGGGACCGGCTACCAACCCCAACA |

1. **N-terminal and C-termina**l deletion mutants:

| 5’HCF-1_N1011 Δ451-750_ | CACGCTCCTGCCCCACCTGGGCATCAGCAG |
| --- | --- |
| 3’HCF-1_N1011 Δ451-750_ | CTGCTGATGCCCAGGTGGGGCAGGAGCGTG |
| 5’HCF-1 _N1011 Δ451-700_ | CACGCTCCTGCCCCCTGGGGCAGGAGCGTG |
| 3’HCF-1 _N1011 Δ451-700_ | CACGCTCCTGCCCCAGGGGGCAGGAGCGTG |
| 5’HCF-1 _N1011 Δ451-650_ | CACGCTCCTGCCCCGACCACACAGTTGTGGG |
| 3’HCF-1 _N1011 Δ451-650_ | CCCACAACTGTGTGGTCGGGGCAGGAGCGTG |
| 5’HCF-1 _N1011 Δ451-550_ | CACGCTCCTGCCCCACGCTGCGGCCGCTGC |
| 3’HCF-1 _N1011 Δ451-550_ | GCAGCGGCCGCAGCGTGGGGCAGGAGCGTG |
| 5’HCF-1 _N1011 Δ751-1000_ | GACCAAGCCCACCATCCAGGGTGATGTGCA |
| 3’HCF-1 _N1011 Δ751-1000_ | TGCACATCACCCTGGATGGTGGGCTTGGTC |
| 5’HCF-1 _N1011 Δ801-1000_ | CATCACCATCACACCCAGGGTGATGTGCA |
| 3’HCF-1 _N1011 Δ801-1000_ | TGCACATCACCCTGGGTGTGATGGTGATG |
| 5’HCF-1 _N1011 Δ851-1000_ | CATCCTCCGCACTGTCCAGGGTGAATGTGCA |
| 3’HCF-1 _N1011 Δ851-1000_ | TGCACATTCACCCTGGACAGTGCGGAGGATG |
| 5’HCF-1 _N1011 Δ901-1000_ | CGGGGCGGGGGGCCACCAGGGTGATGTGCA |
| 3’HCF-1 _N1011 Δ901-1000_ | TGCACATCACCCTGGTGGCCCCCCGCCCCG |

1. **HCF-1 duplication mutants and other constructs**:

| 5’HCF-1 _N1011_ D1 | GATCCAGGCTGCCCCCGCACCCCCG |
| --- | --- |
| 3’HCF-1 _N1011_ D1 | CGGGGGTGCGGGGGCAGCCTGGATC |
| 5’HCF-1 _N1011_ D2 | GATCGCGTCCACGGGTCCTGTGAC |
| 3’HCF-1 _N1011_ D2 | GTCACAGGACCCGTGGACGCGATC |
| 5’HCF-1 _N1011 Δ Basic_ | CTGCACATCACCCTGTGGGGCAGGAGCGTG |
| 3’HCF-1 _N1011 Δ Basic_ | CACGCTCCTGCCCCACAGGGTGATGTGCAG |
| 5’HCF-1 _N1011_ D1-D1 STOP | TGCGGGGGCAGCCTGCACCAGCTTCAGGAT |
| 3’HCF-1 _N1011_ D1-D1 STOP | ATCCTGAAGCTGGTGCAGGCTGCCCCCGCA |
| 5’HCF-1 _N1011_ D2-D2 STOP | GCCATCTGCTGAGGTGCCTGAGTCGGCGAT |
| 3’HCF-1 _N1011_ D2-D2 STOP | ATCGCCGACTCAGGCACCTCAGCAGATGGC |
