# Supplementary material for: Role of the HCF-1 Basic Region in Sustaining Cell Proliferation
Source: PLoS One. 2010 Feb 2;5(2):e9020. doi: 10.1371/journal.pone.0009020 (PMC2814863; doi:10.1371/journal.pone.0009020)
Supplement: Figure S1 — tsBN67 colony assay with the 11 scanning deletion mutants. (2.40 MB PDF) [file pone.0009020.s002.pdf]

Supplemental Figure 1: tsBN67 colony assay with the 11 scanning deletion mutants

HCF-1$_{N1011}$

1st transfection

2nd transfection

3rd transfection

Permissive

Non Permissive

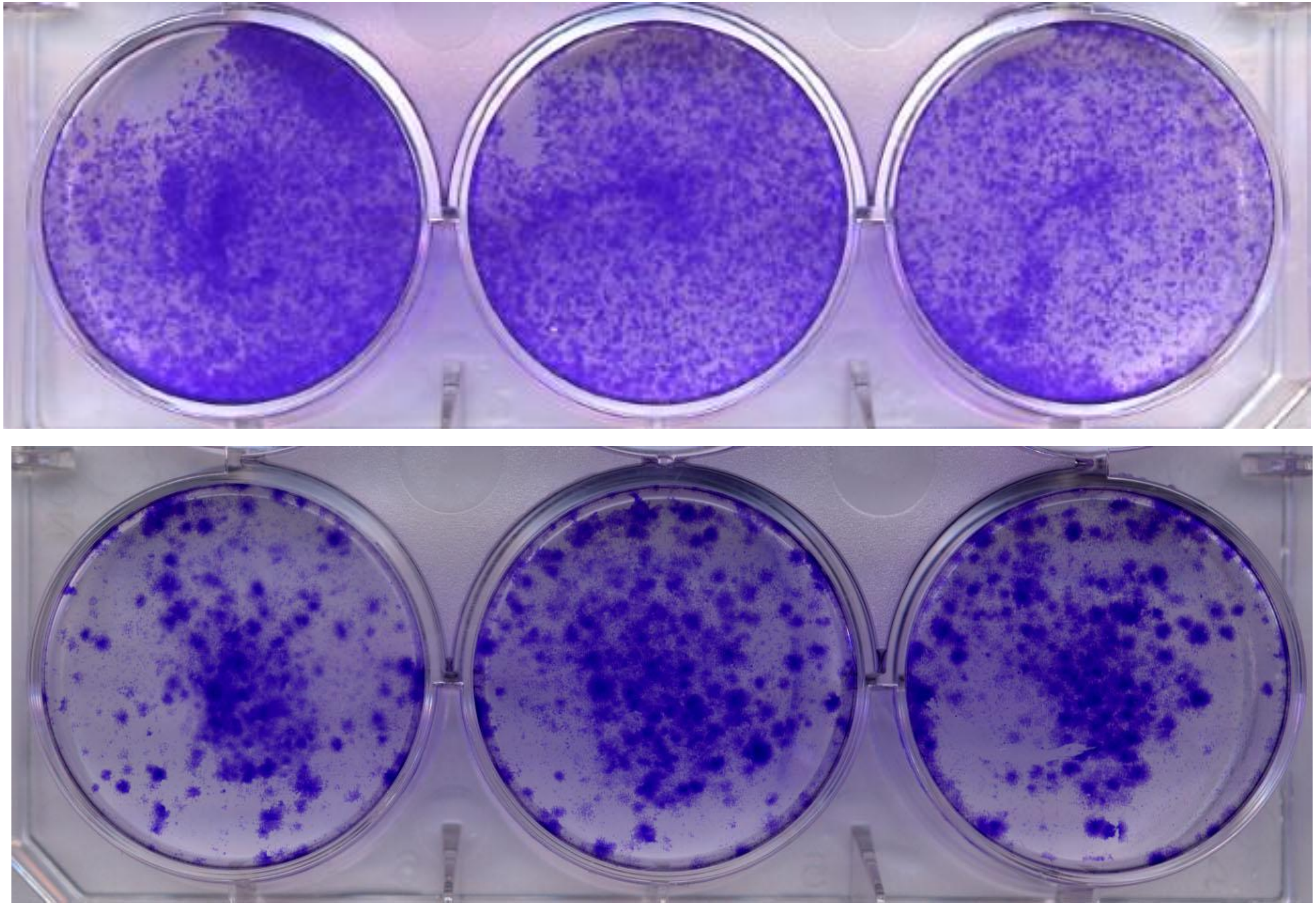

HCF-1$_{N1011\Delta450-500}$

1st transfection | 2nd transfection | 3rd transfection

Permissive

Non Permissive

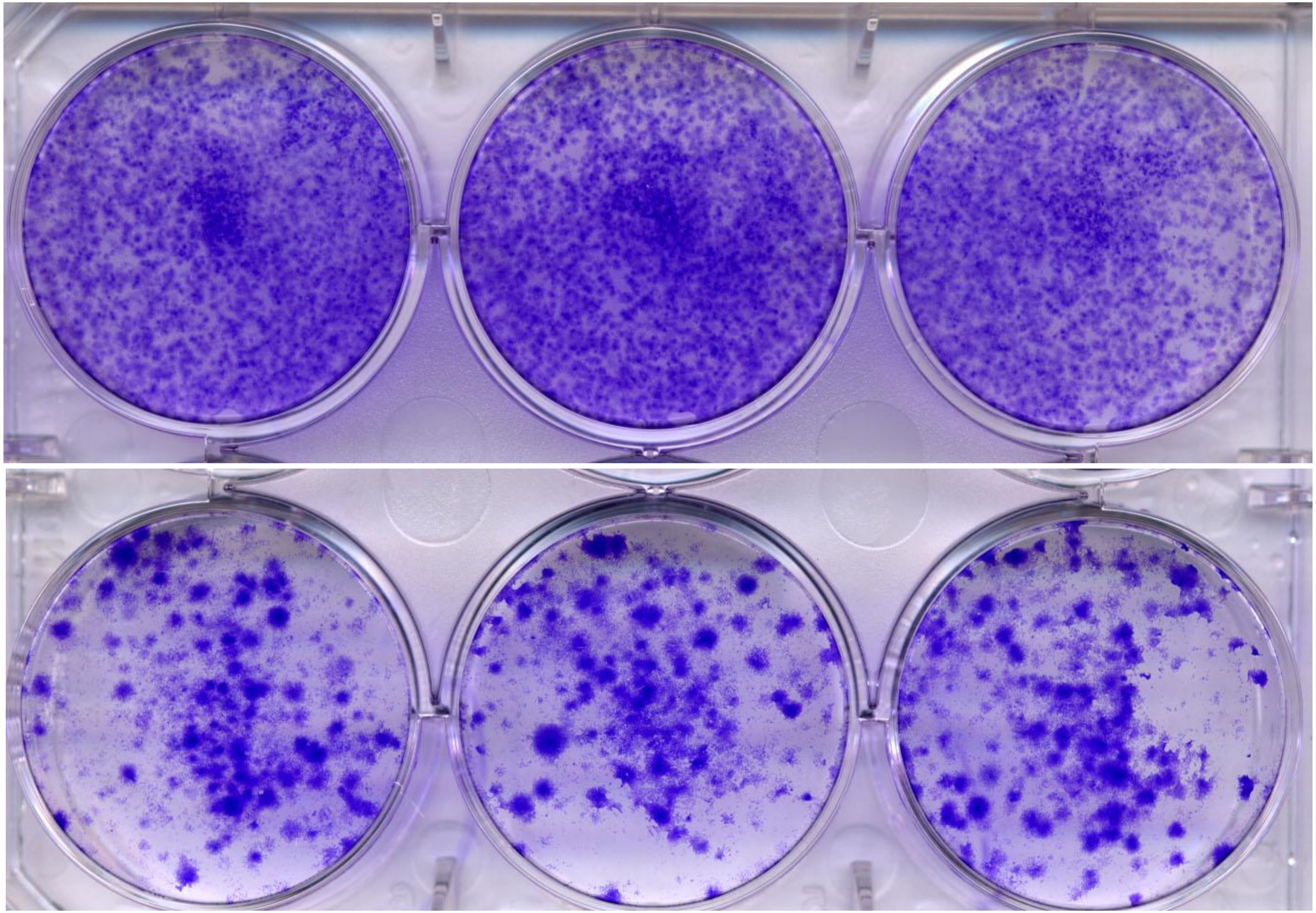

HCF-1$_{N1011\Delta500\text{-}550}$

1st transfection

2nd transfection

3rd transfection

Permissive

Non Permissive

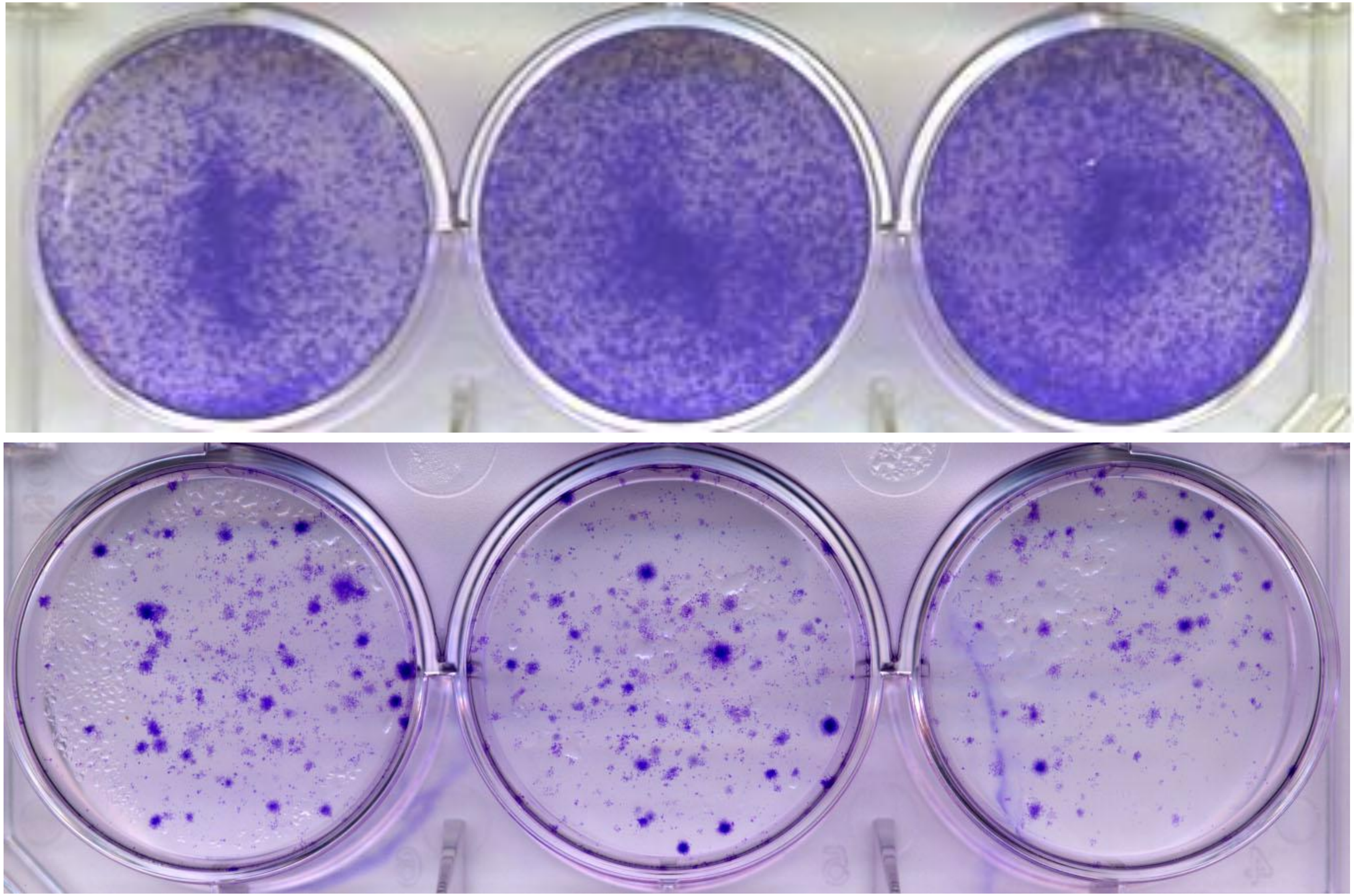

HCF-1$_{N1011\Delta 550\text{-}600}$

1st transfection 2nd transfection 3rd transfection

Permissive

Non Permissive

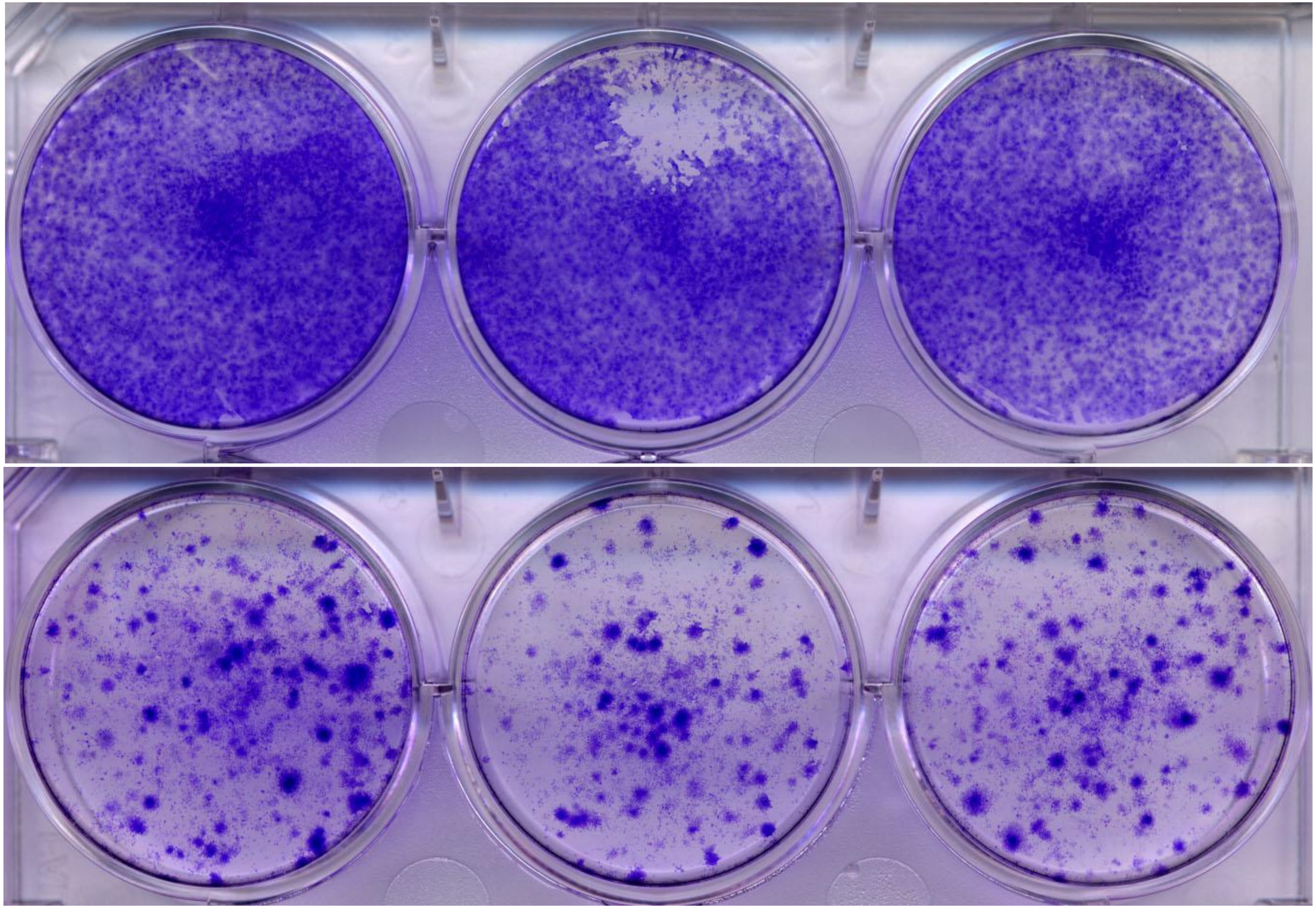

HCF-1$_{N1011\Delta600\text{-}650}$

1st transfection

2nd transfection

3rd transfection

Permissive

Non Permissive

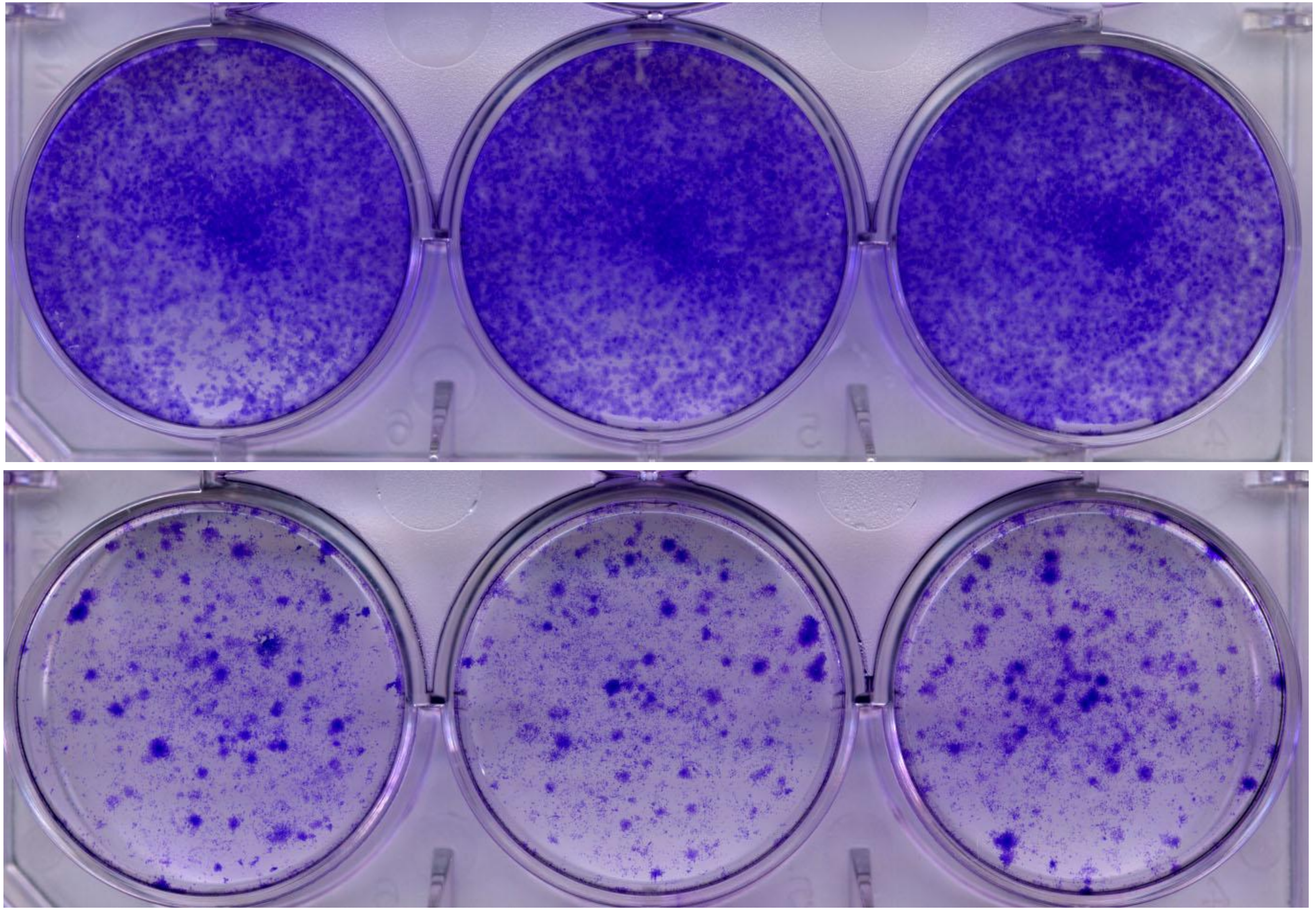

HCF-1$_{N1011\Delta 650\text{-}700}$

1st transfection 2nd transfection 3rd transfection

Permissive

Non Permissive

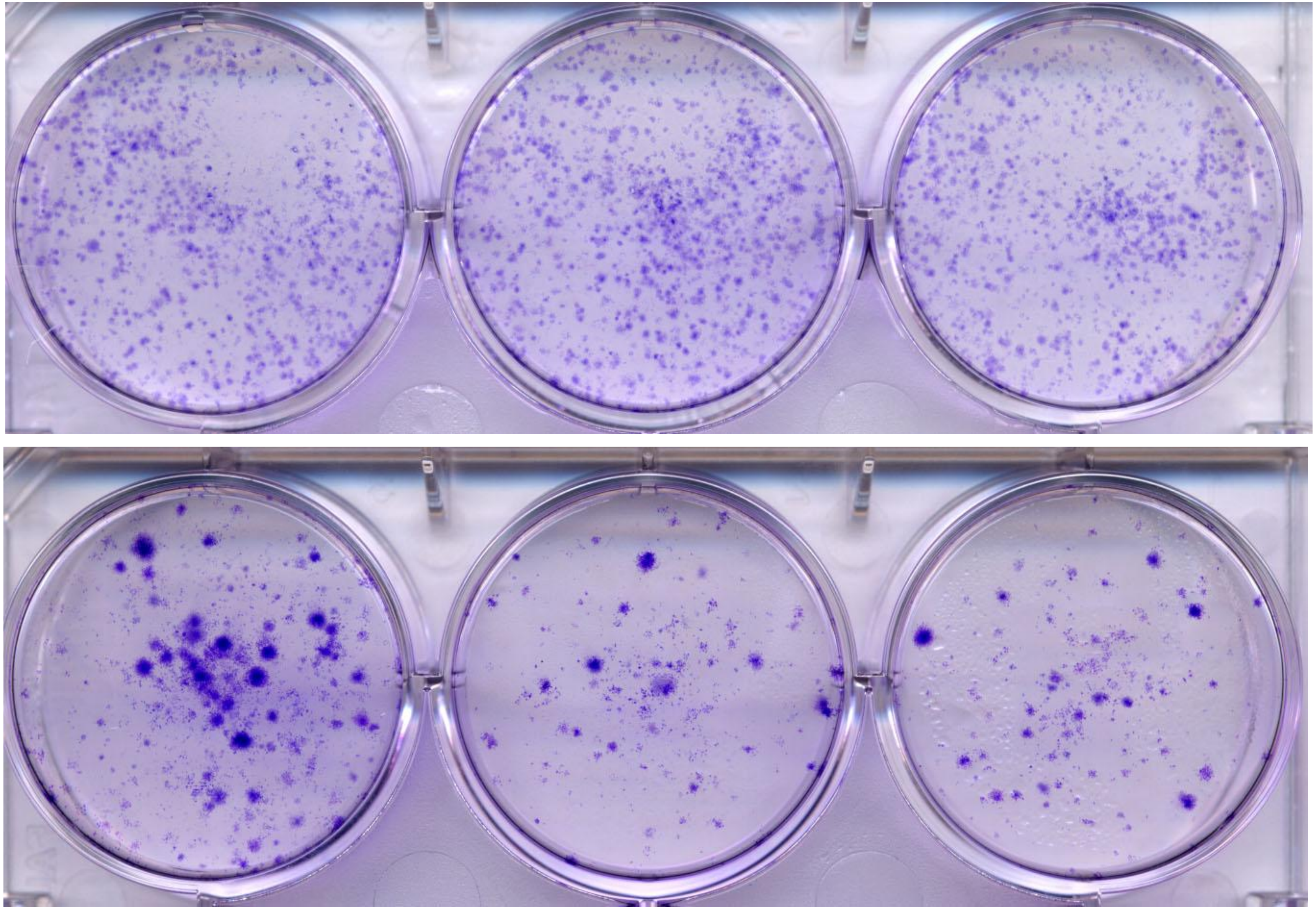

HCF-1$_{N1011\Delta700\text{-}750}$

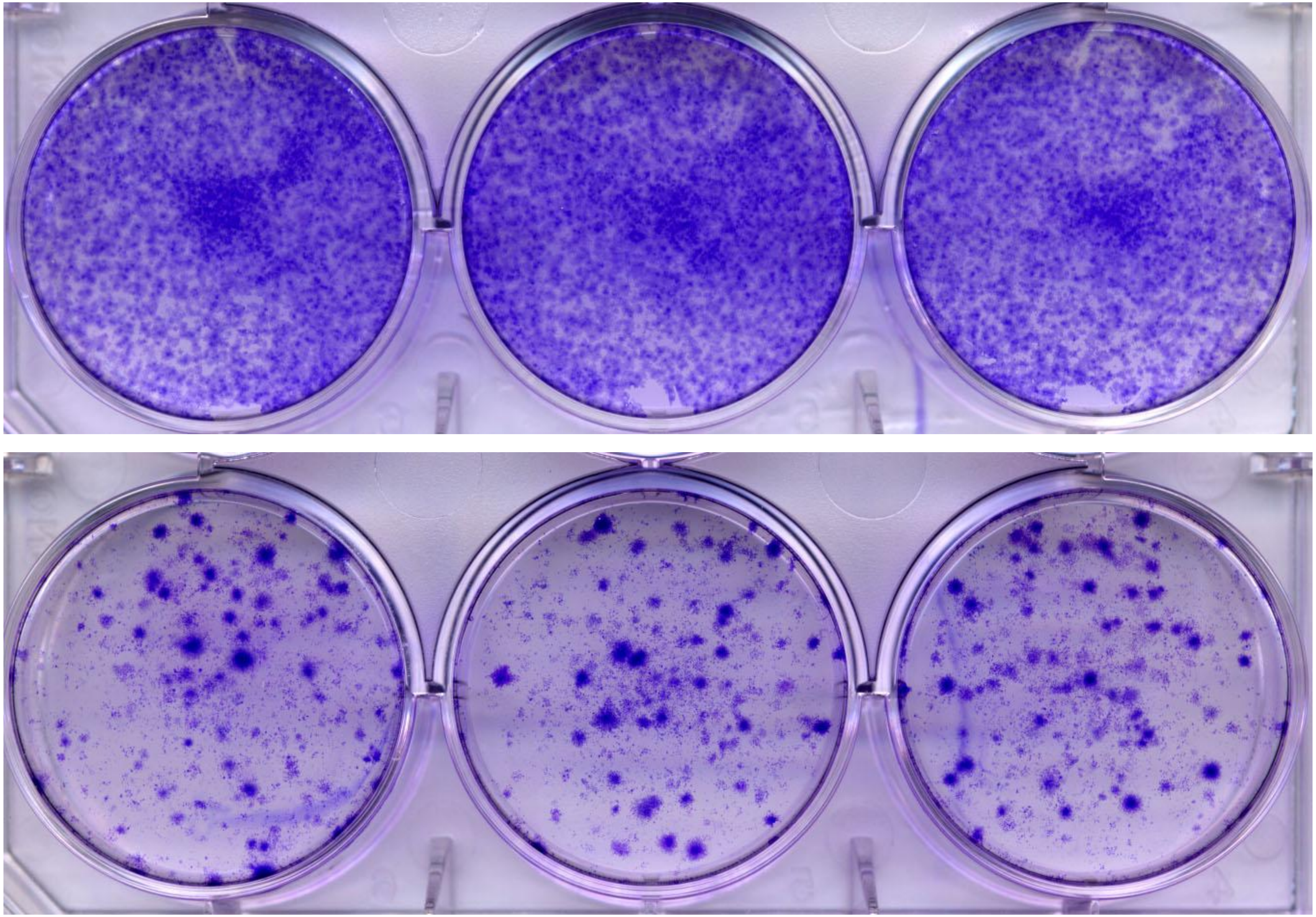

HCF-1$_{N1011\Delta750\text{-}800}$

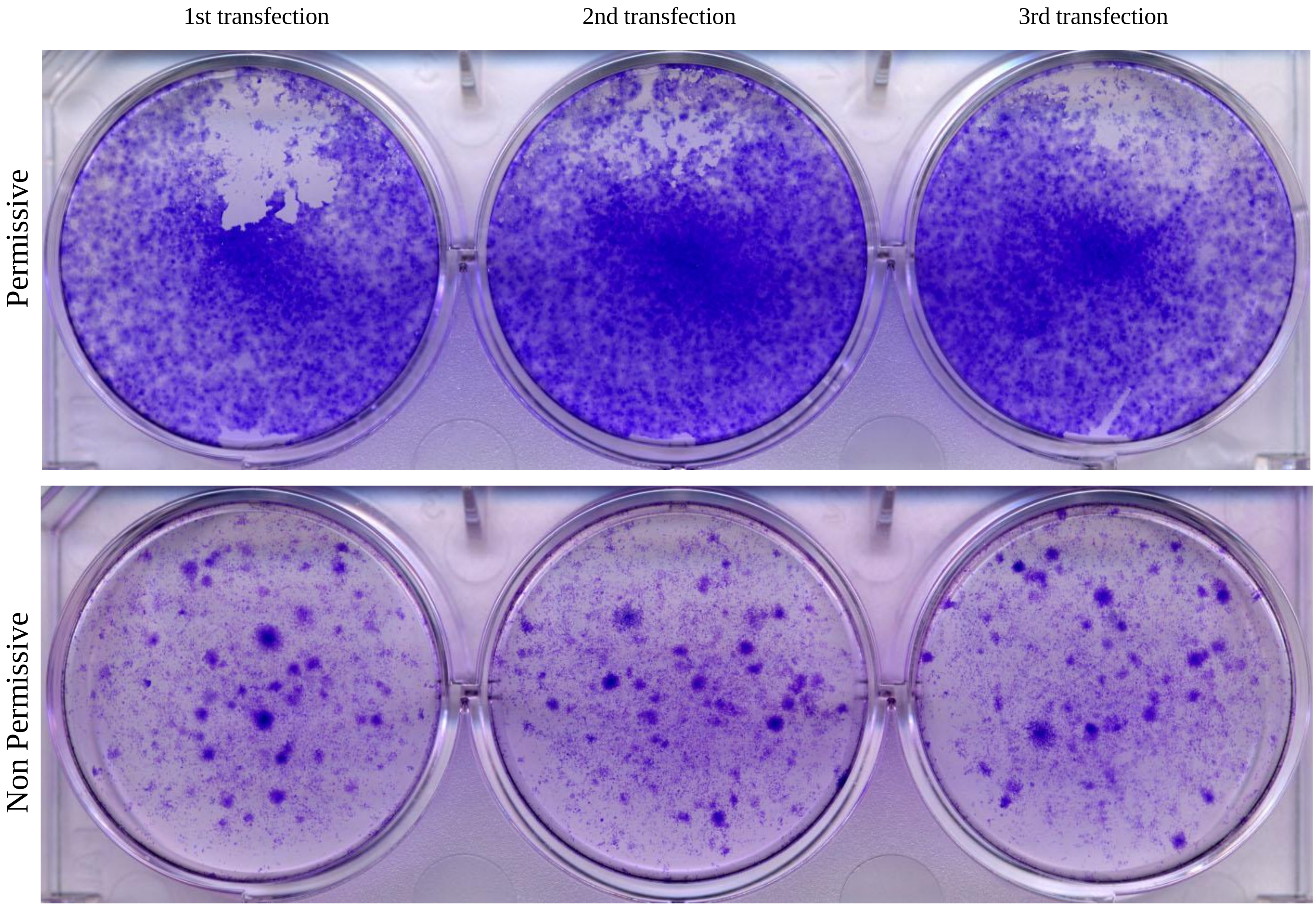

HCF-1$_{N1011\Delta800\text{-}850}$

1st transfection | 2nd transfection | 3rd transfection

Permissive

Non Permissive

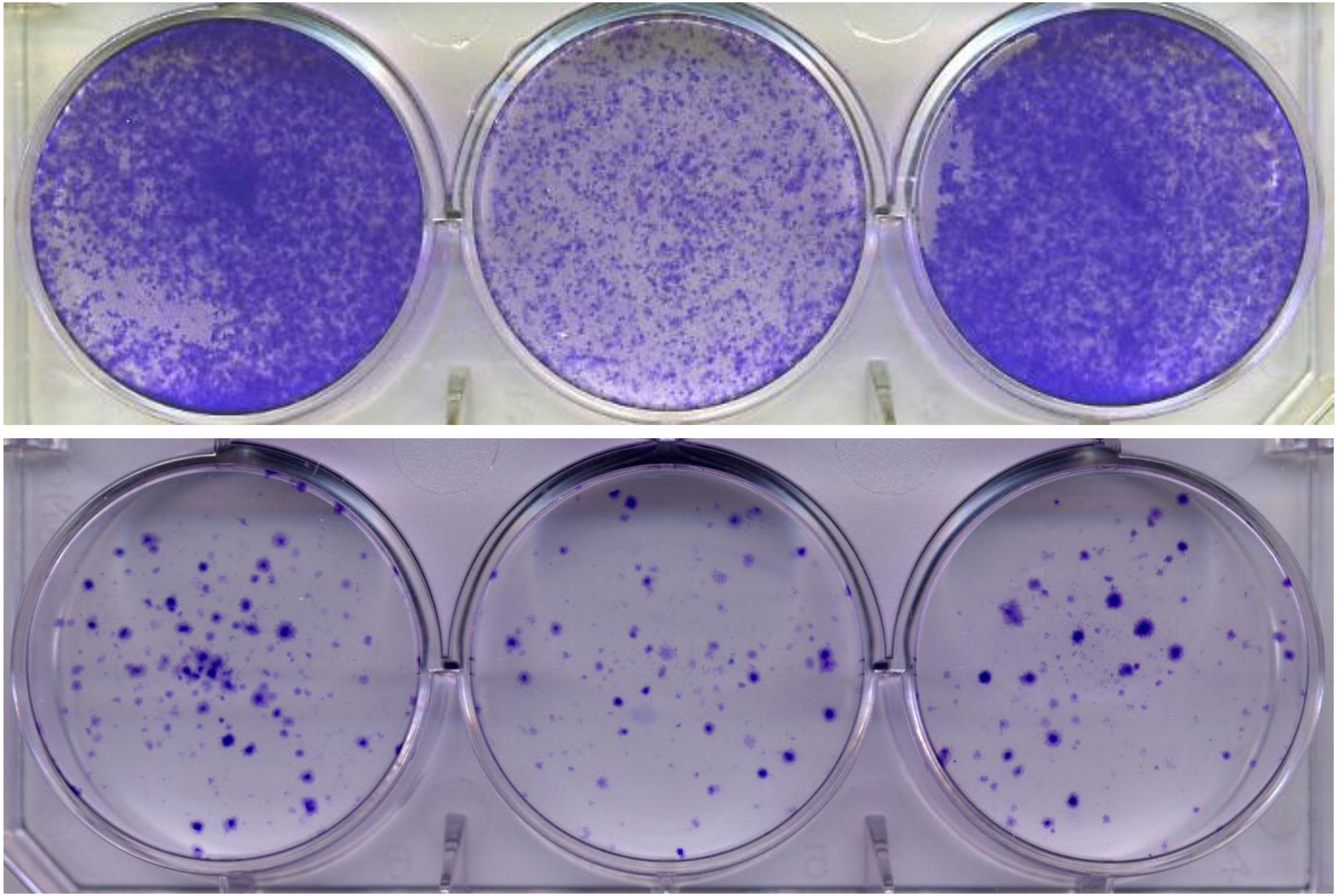

HCF-$1_{N1011\Delta850\text{-}900}$

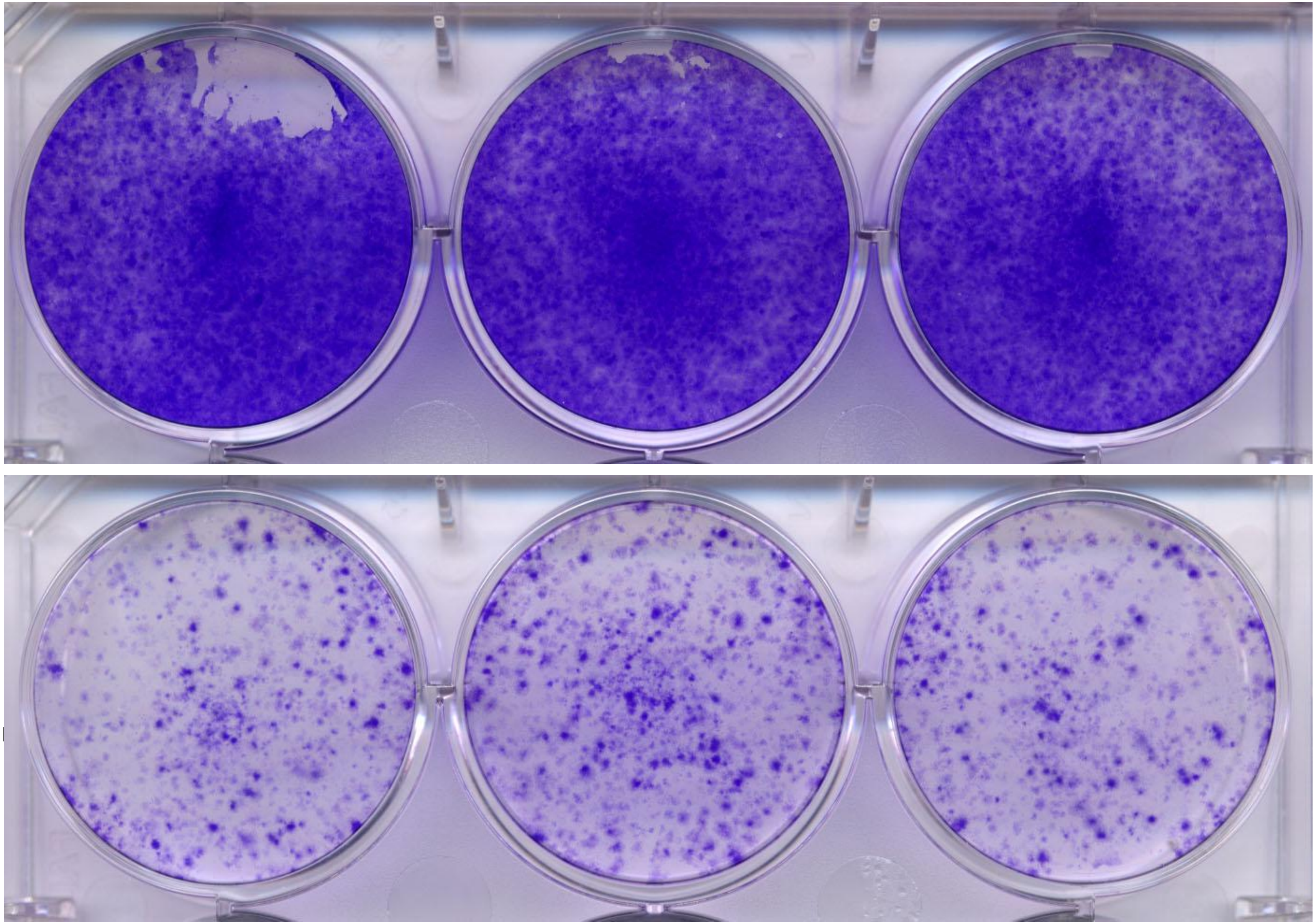

HCF-1$_{N1011\Delta900\text{-}950}$

1st transfection | 2nd transfection | 3rd transfection

Permissive

Non Permissive

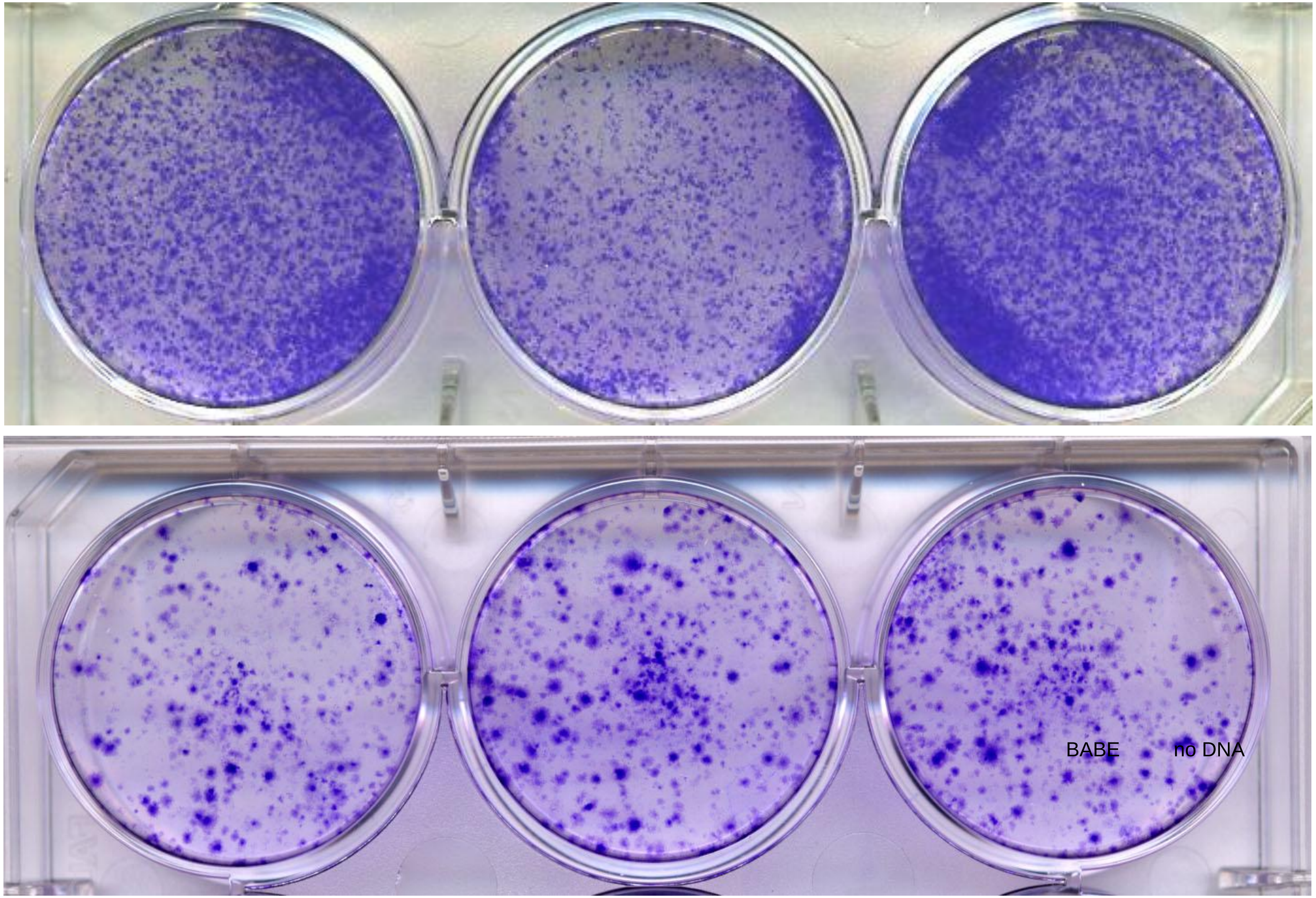

HCF-1$_{N1011\Delta 950\text{-}1000}$

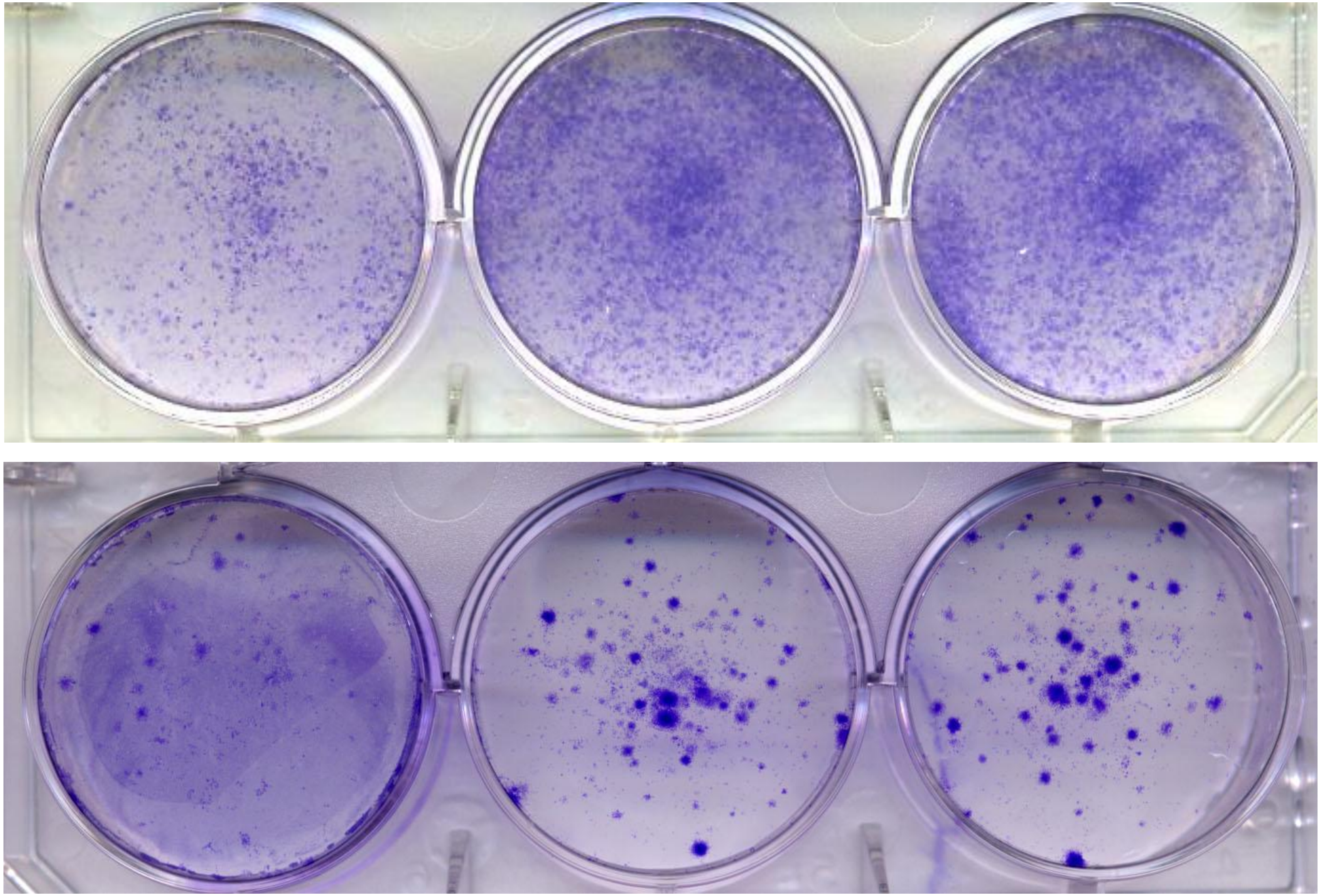

HCF-1$_{N1011\Delta451\text{-}1000}$

1st transfection 2nd transfection 3rd transfection

Permissive

Non Permissive

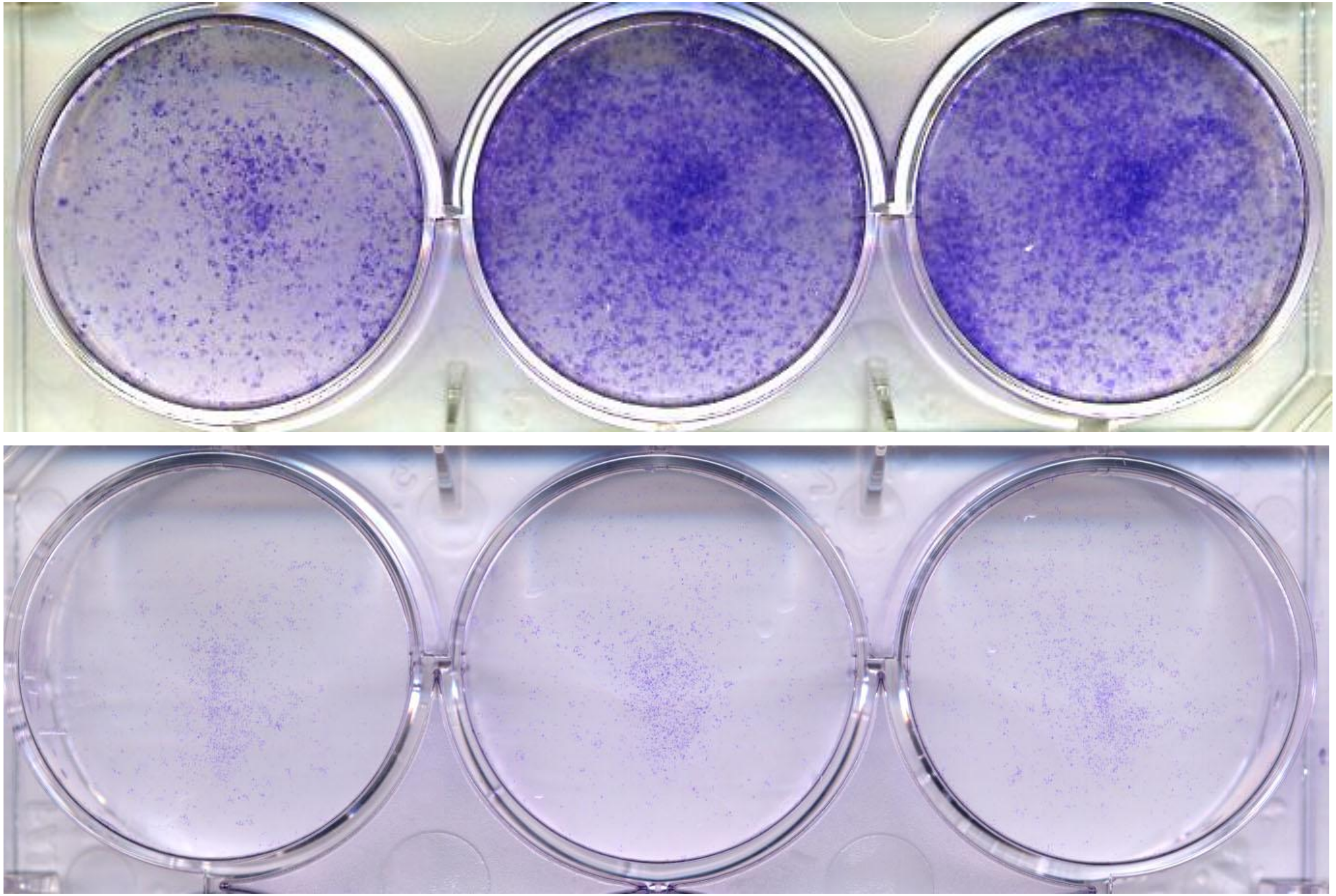

HCF-1$_{N1011\Delta381\text{-}1000}$

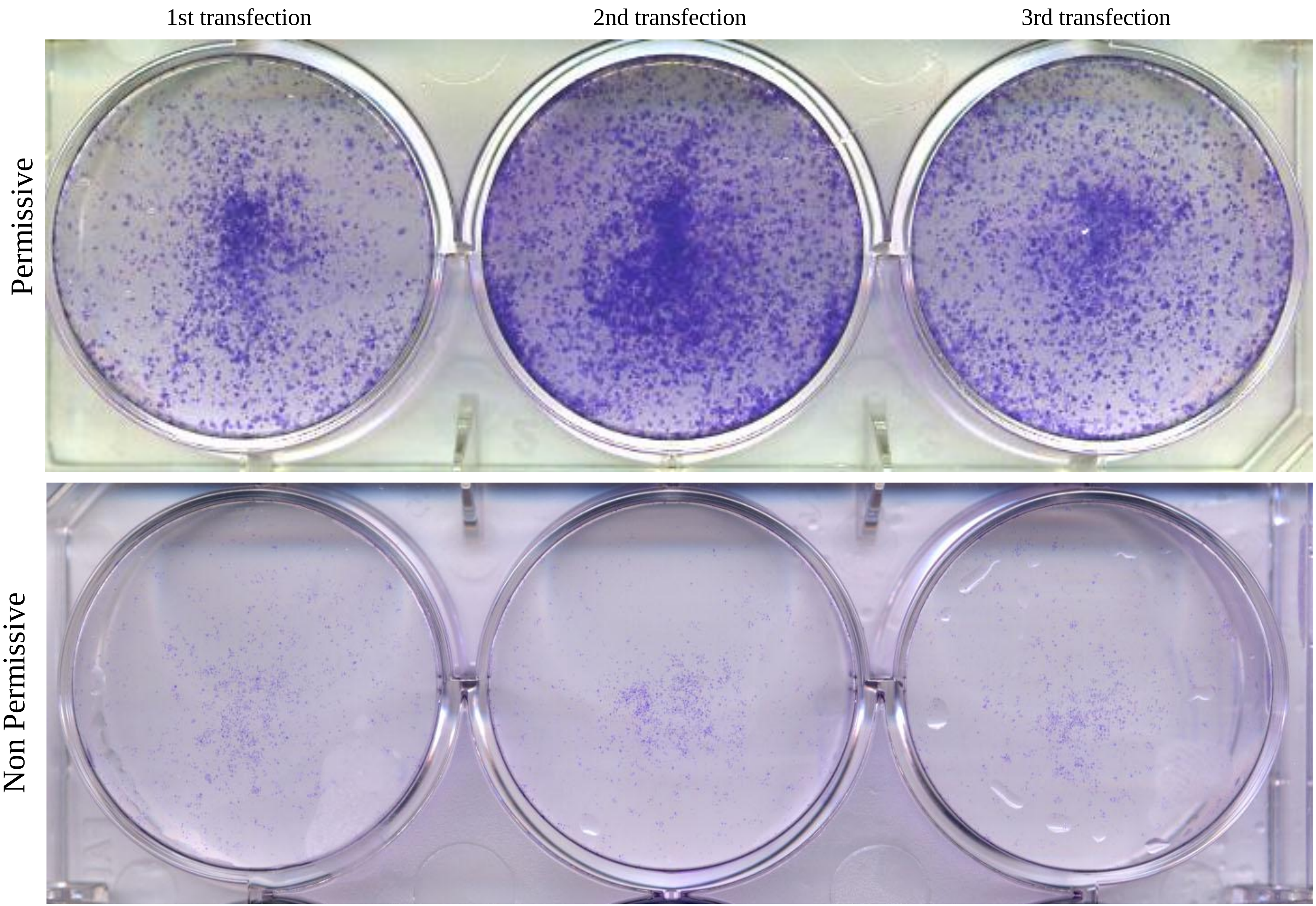

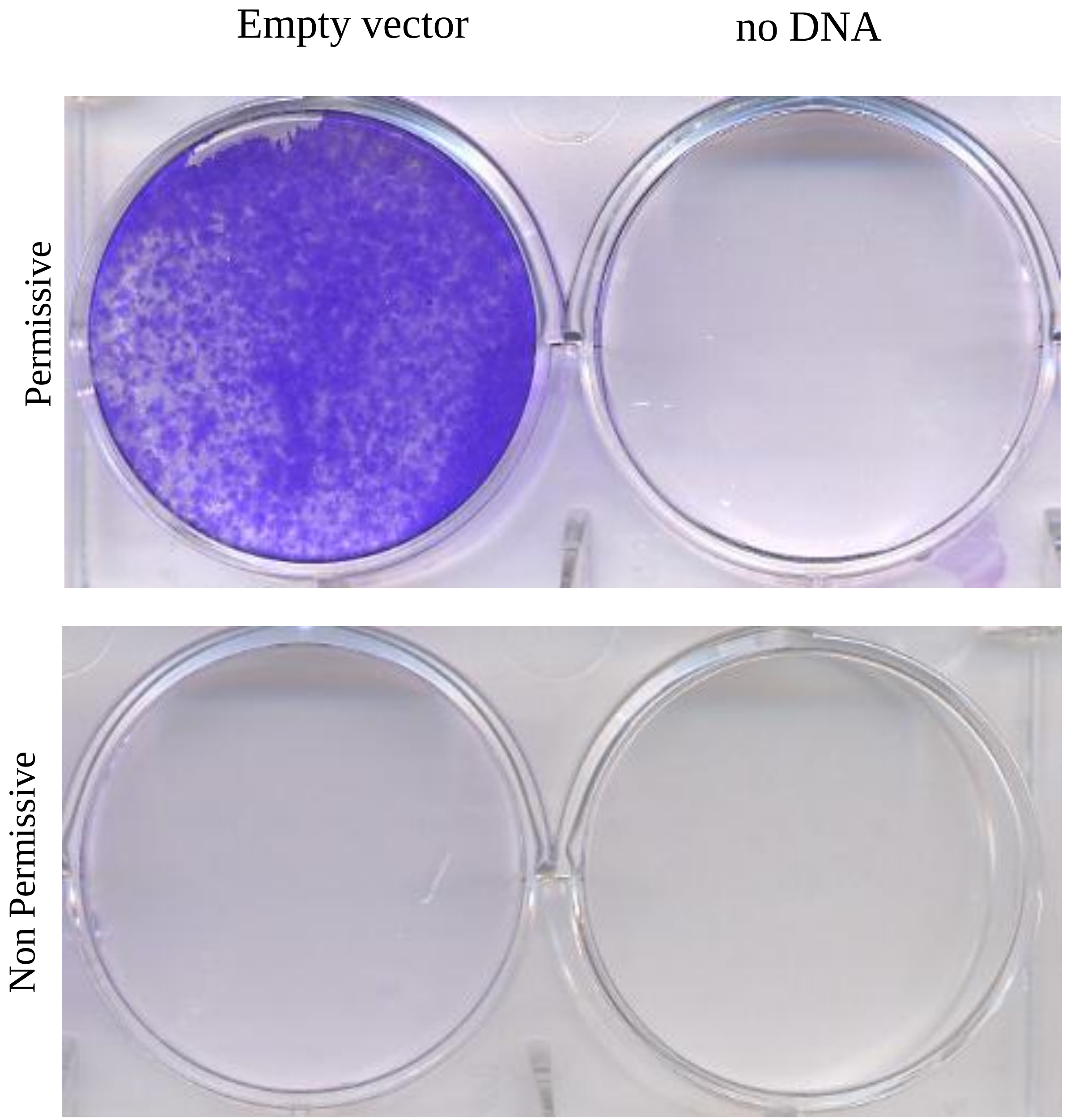
Empty vector
no DNA
Permissive
Non Permissive
