# Supplementary material for: Role of the HCF-1 Basic Region in Sustaining Cell Proliferation
Source: PLoS One. 2010 Feb 2;5(2):e9020. doi: 10.1371/journal.pone.0009020 (PMC2814863; doi:10.1371/journal.pone.0009020)
Supplement: Figure S2 — tsBN67 colony assay with the N-and C-terminal deletion mutants. (2.12 MB PDF) [file pone.0009020.s003.pdf]

Supplemental Figure 2:
tsBN67 colony assay with the N- and C-terminal deletion mutants

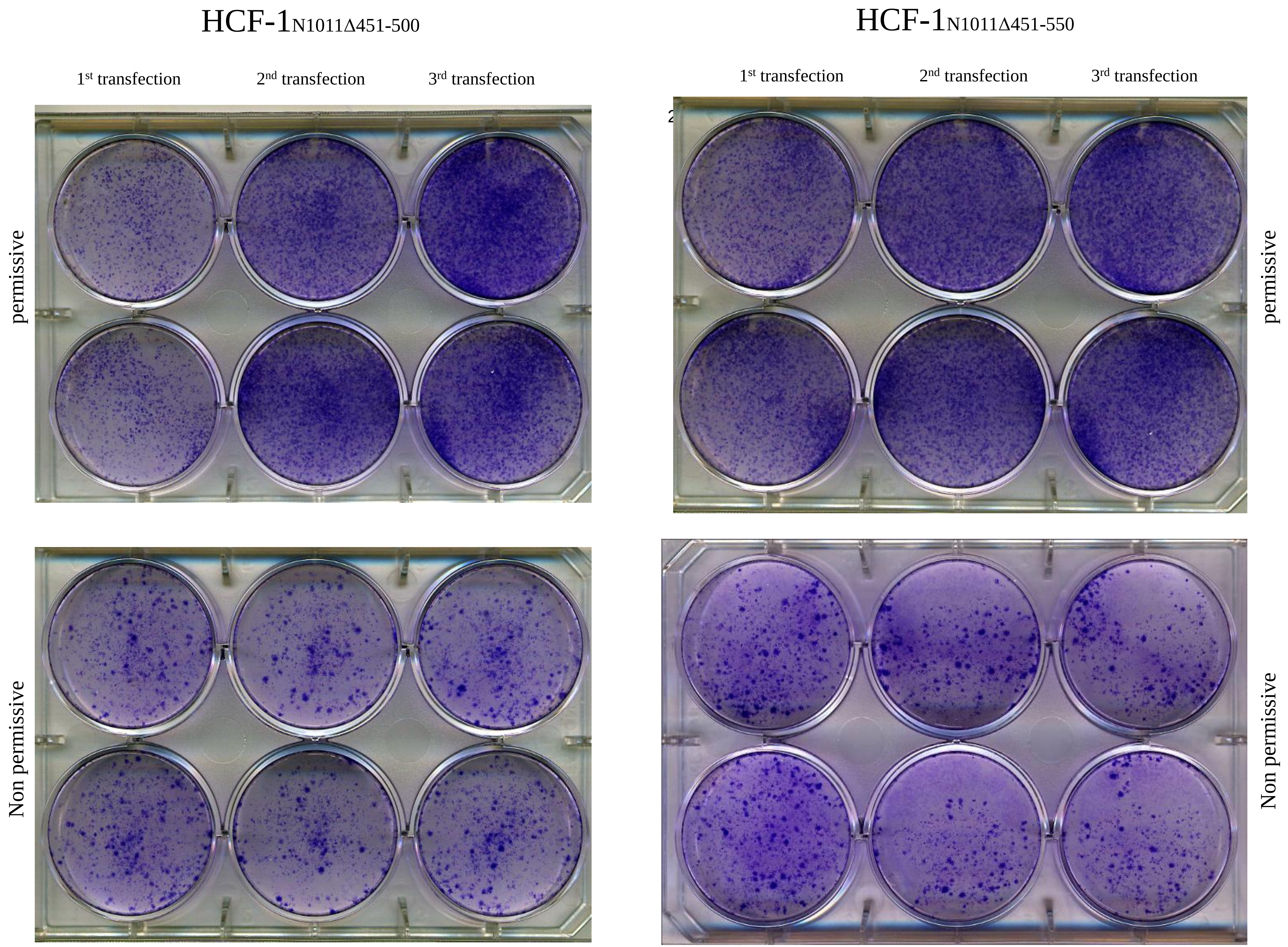
HCF-1N1011Δ451-500
HCF-1N1011Δ451-550
1st transfection
2nd transfection
3rd transfection
1st transfection
2nd transfection
3rd transfection
permissive
permissive
Non permissive
Non permissive

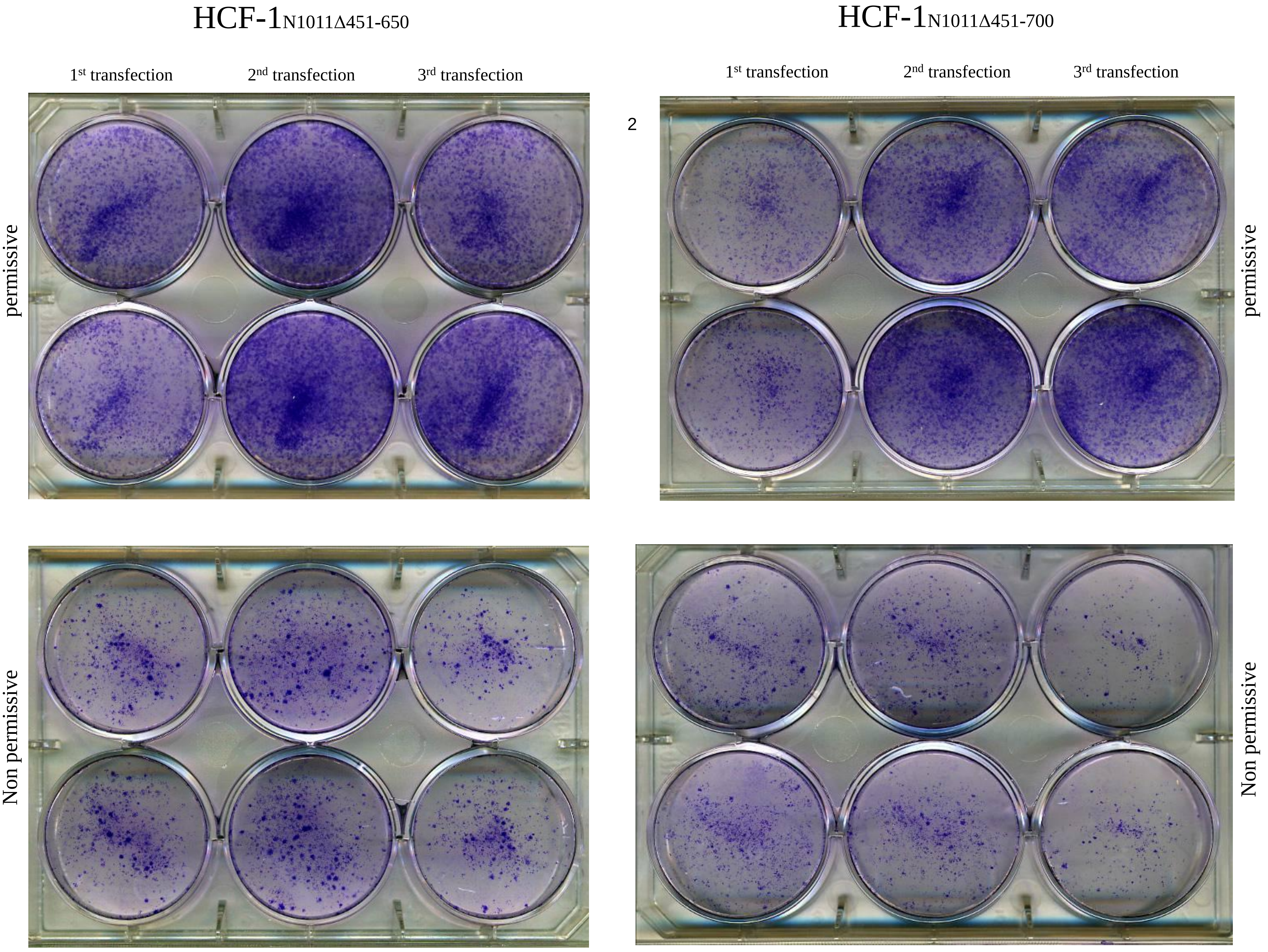

$\text{HCF-1}_{\text{N1011}\Delta\text{451-650}}$
$\text{HCF-1}_{\text{N1011}\Delta\text{451-700}}$
1st transfection
2nd transfection
3rd transfection
1st transfection
2nd transfection
3rd transfection
2
permissive
permissive
Non permissive
Non permissive

HCF-1$_{N1011\Delta451\text{-}750}$

1st transfection | 2nd transfection | 3rd transfection

permissive

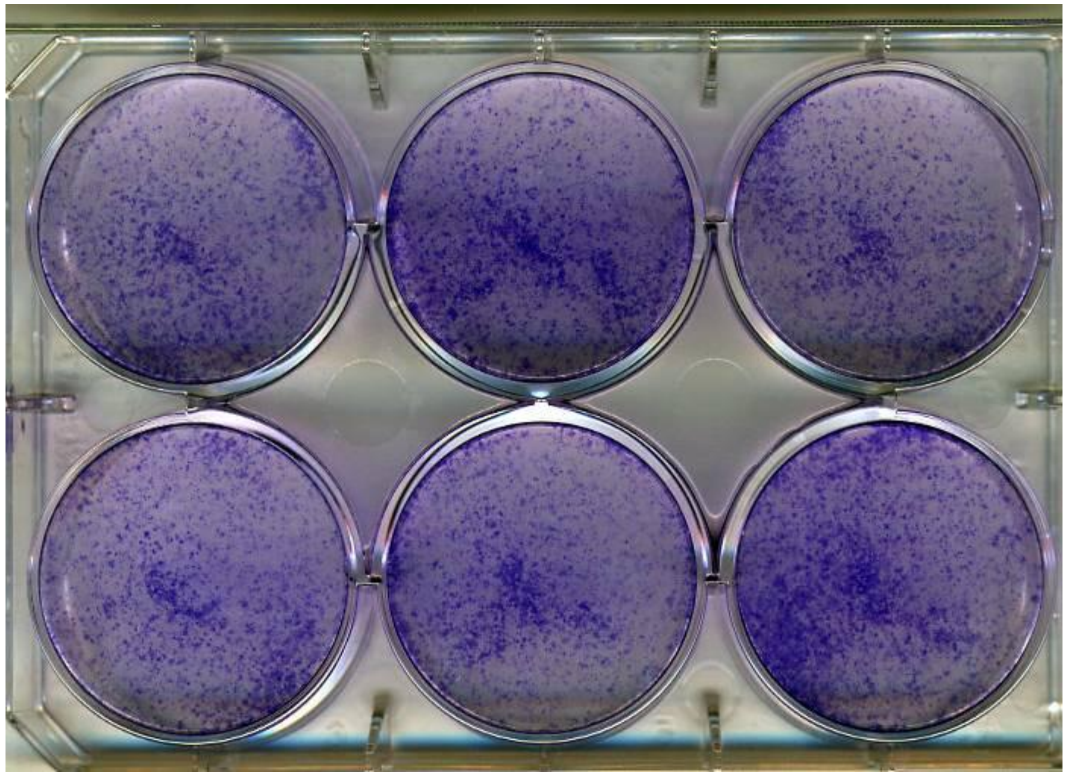

Non permissive

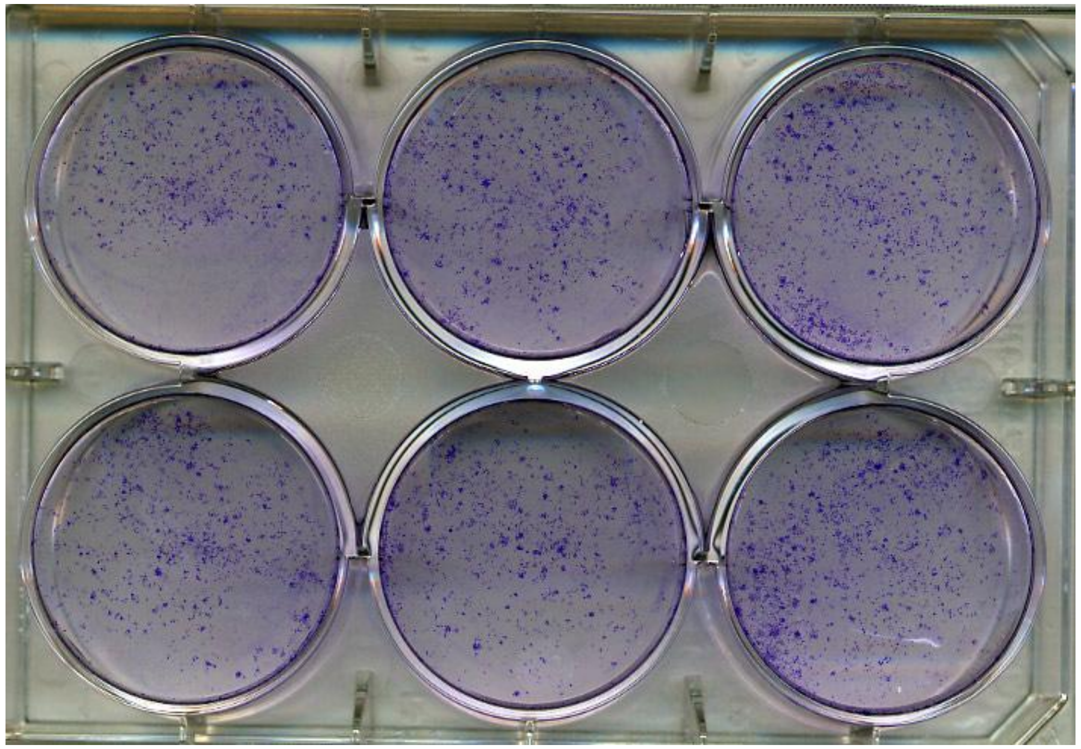

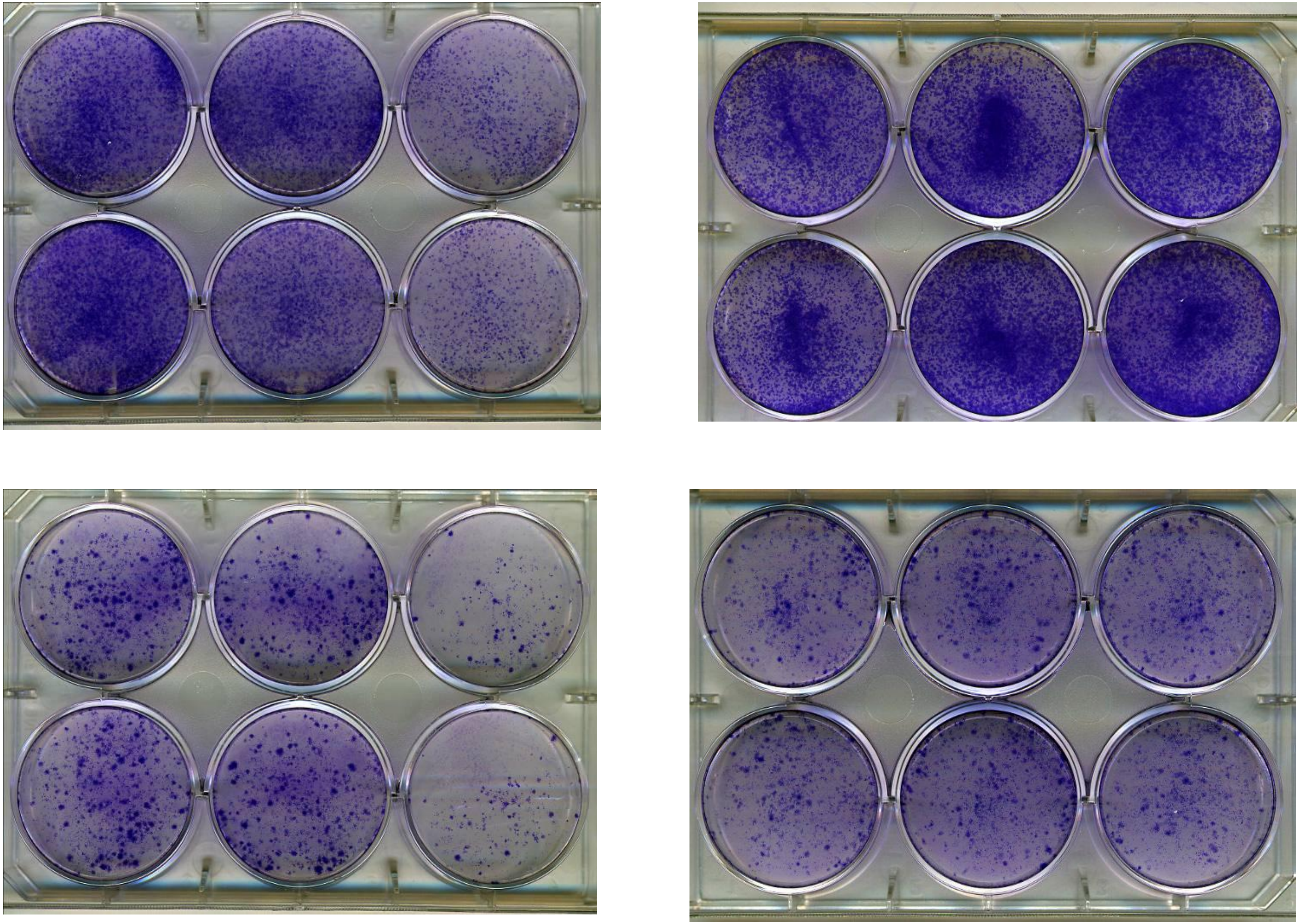
$HCF\text{-}1_{N1011\Delta 951\text{-}1000}$
$HCF\text{-}1_{N1011\Delta 901\text{-}1000}$
1st transfection
2nd transfection
3rd transfection
1st transfection
2nd transfection
3rd transfection
permissive
permissive
Non permissive
Non permissive

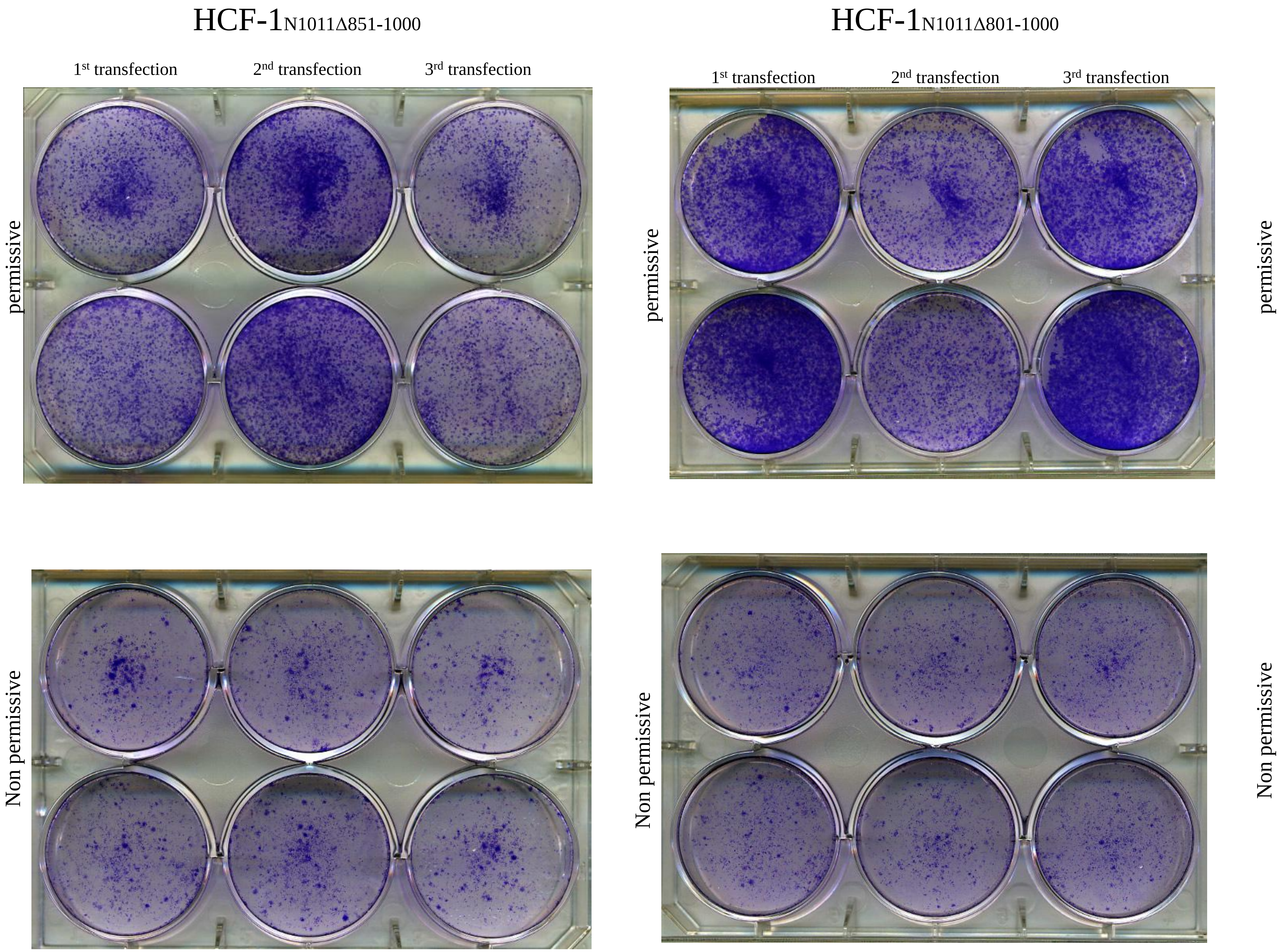
HCF-1$_{N1011\Delta851-1000}$
HCF-1$_{N1011\Delta801-1000}$
1st transfection
2nd transfection
3rd transfection
1st transfection
2nd transfection
3rd transfection
permissive
permissive
permissive
Non permissive
Non permissive
Non permissive

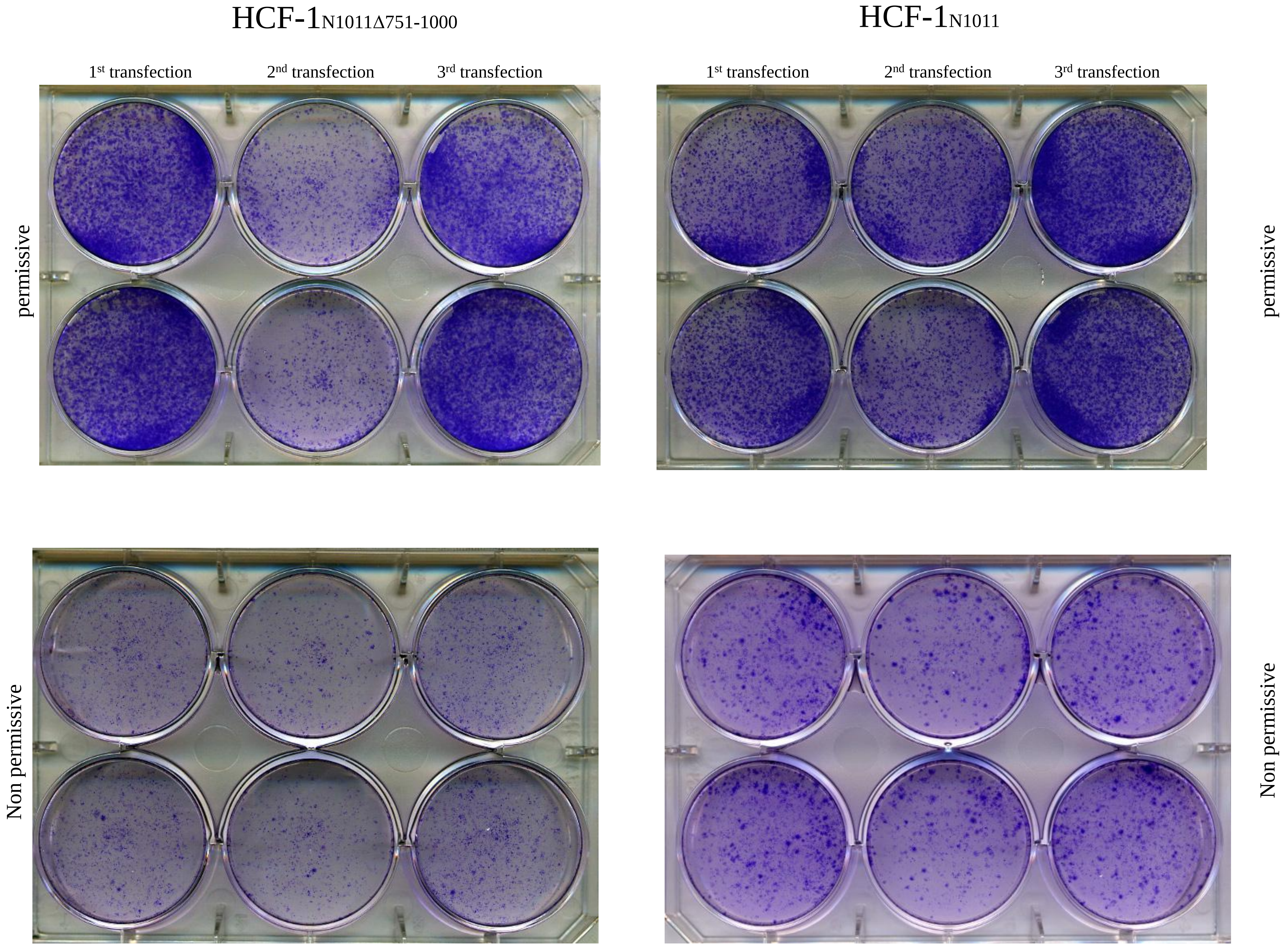
HCF-1N1011Δ751-1000
HCF-1N1011
1st transfection
2nd transfection
3rd transfection
1st transfection
2nd transfection
3rd transfection
permissive
permissive
Non permissive
Non permissive

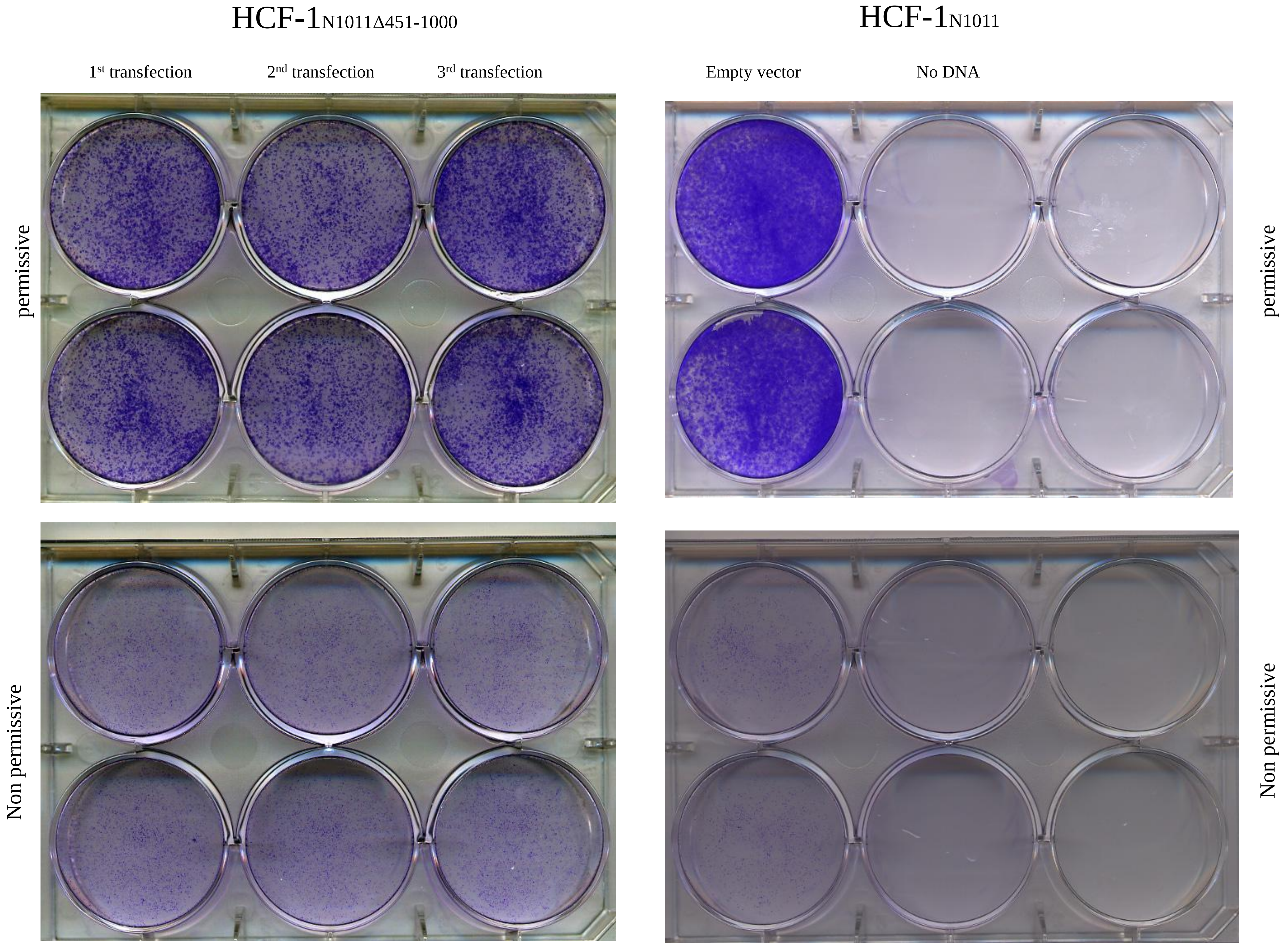
HCF-1N1011Δ451-1000
HCF-1N1011
1st transfection
2nd transfection
3rd transfection
Empty vector
No DNA
permissive
Non permissive
permissive
Non permissive

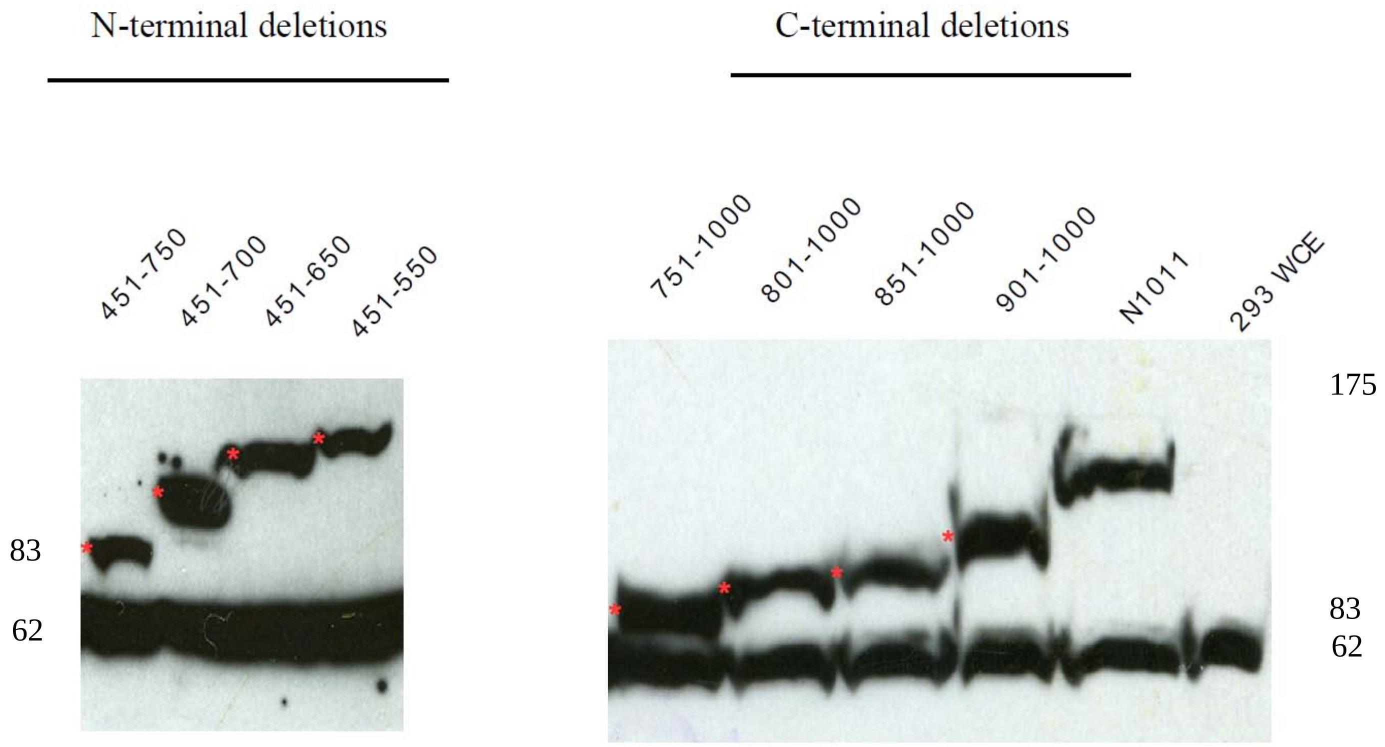

Supplemental Figure 2: Protein expression of the mutants containing increased N- and C-terminal deletions

Each of the N- and C-terminal deletion mutant was tested for protein expression in 293 cells using alpha-HA (12CA5) antibody. All the full length and mutant constructs directed synthesis of an appropriately sized protein. Red asterisks highlight cross-reactive bands for the predicted protein.
