# Supplementary material for: Role of the HCF-1 Basic Region in Sustaining Cell Proliferation
Source: PLoS One. 2010 Feb 2;5(2):e9020. doi: 10.1371/journal.pone.0009020 (PMC2814863; doi:10.1371/journal.pone.0009020)
Supplement: Figure S3 — tsBN67 colony assay with the duplication and deletion mutants. (1.86 MB PDF) [file pone.0009020.s004.pdf]

Supplemental Figure 3:
tsBN67 colony assay with the duplication and deletion mutants

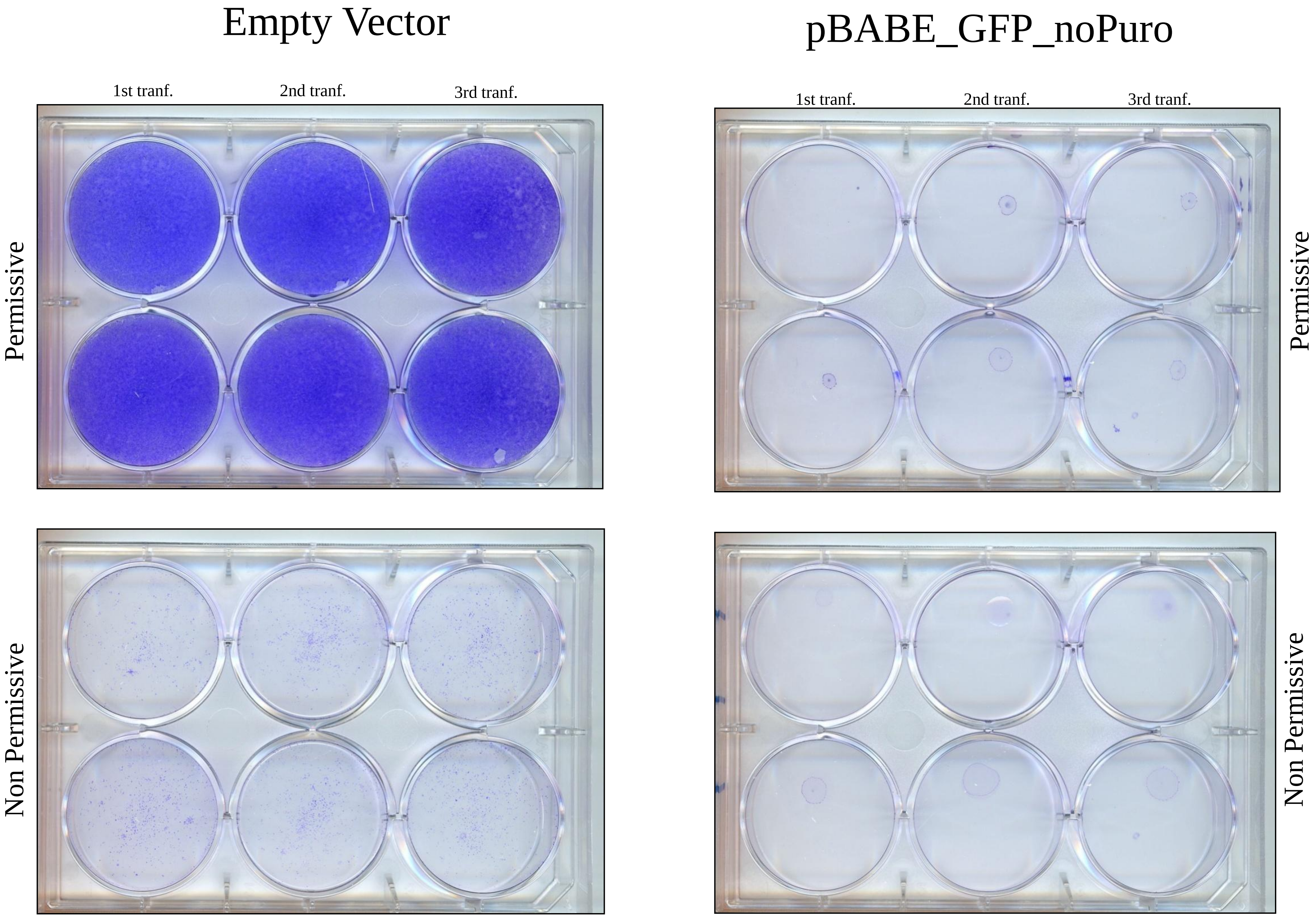
Empty Vector
pBABE_GFP_noPuro
1st tranf.
2nd tranf.
3rd tranf.
1st tranf.
2nd tranf.
3rd tranf.
Permissive
Permissive
Non Permissive
Non Permissive

HCF-1$_{\text{N1011}\Delta\text{381-1000}}$

HCF-1$_{\text{N1011}\Delta\text{451-1000}}$

1st tranf. 2nd tranf. 3rd tranf.

1st tranf. 2nd tranf. 3rd tranf.

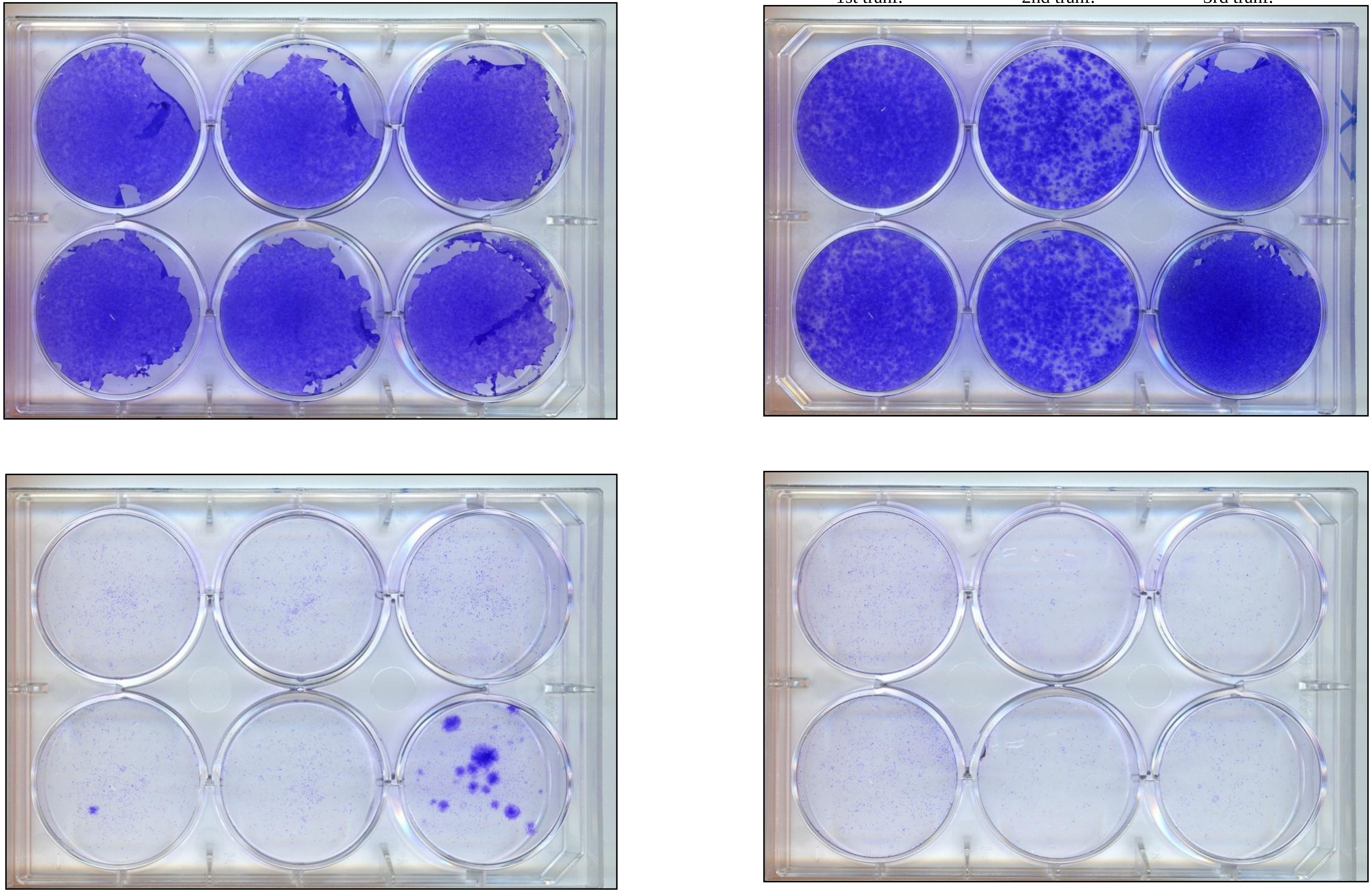

Permissive

Permissive

Non Permissive

Non Permissive

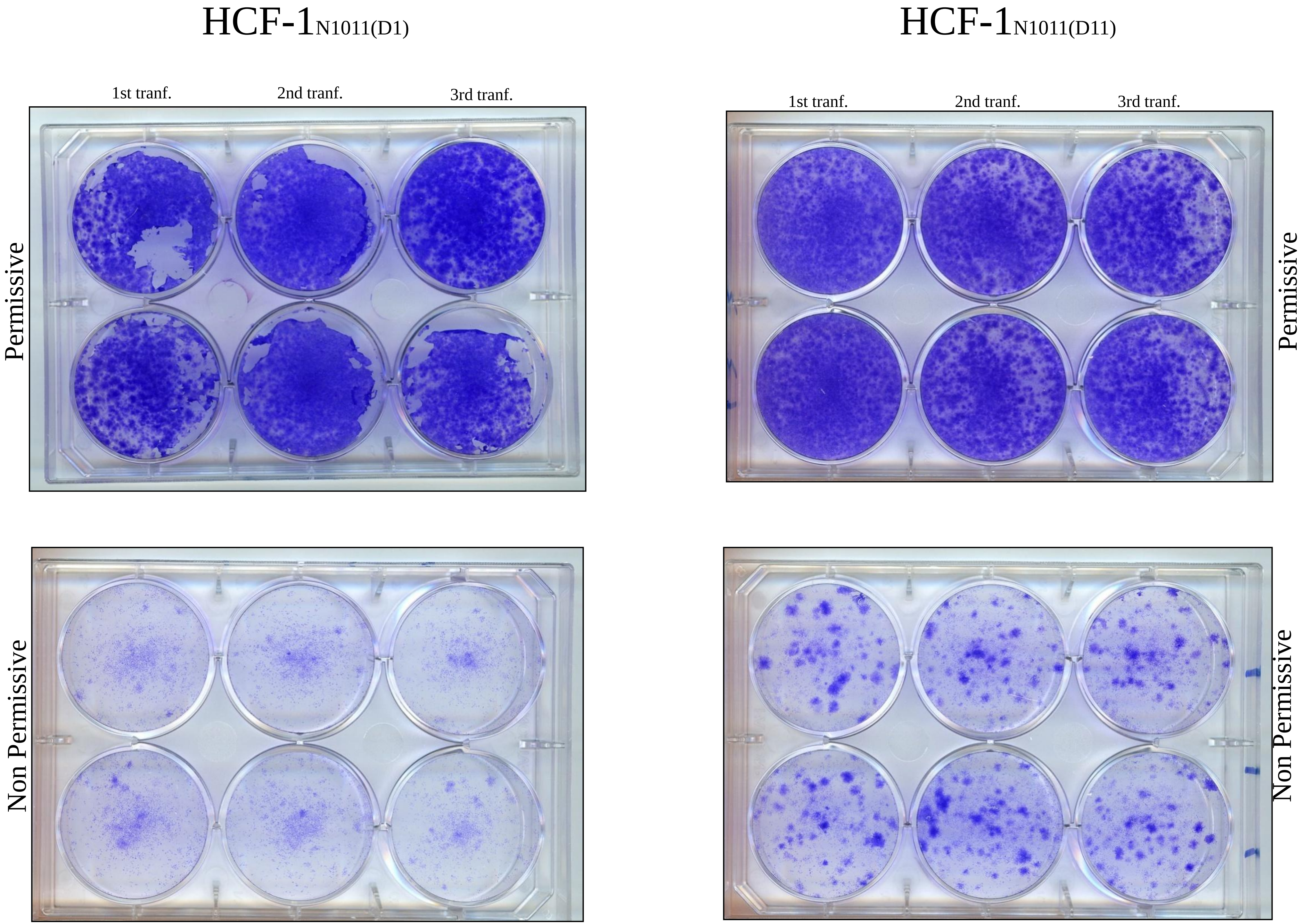
HCF-1N1011(D1)
HCF-1N1011(D11)
1st tranf.
2nd tranf.
3rd tranf.
1st tranf.
2nd tranf.
3rd tranf.
Permissive
Permissive
Non Permissive
Non Permissive

HCF-1 N1011(D2) — HCF-1 N1011(D22)

1st tranf. — 2nd tranf. — 3rd tranf. (repeated for both plates)

Permissive

Non Permissive

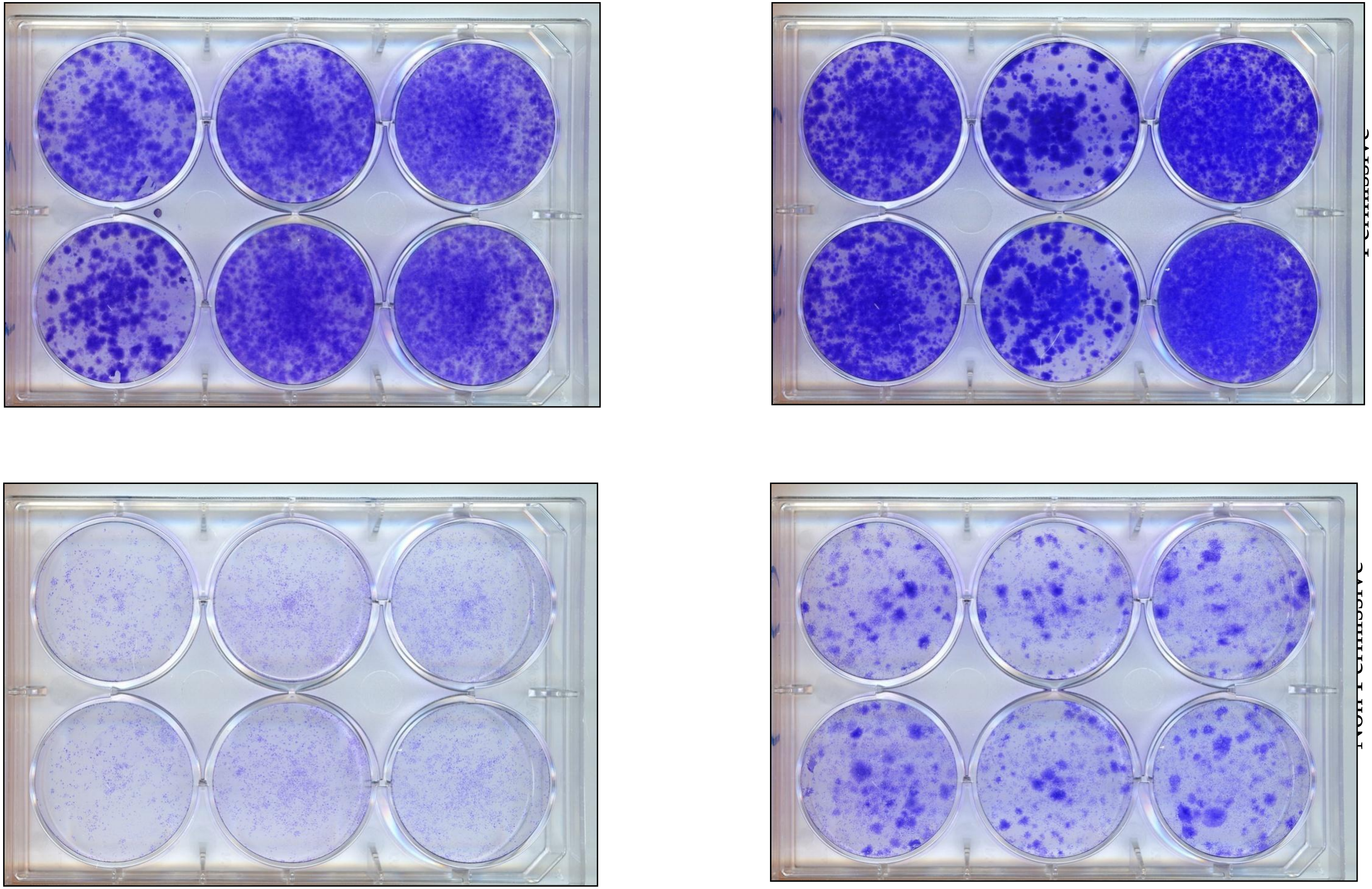

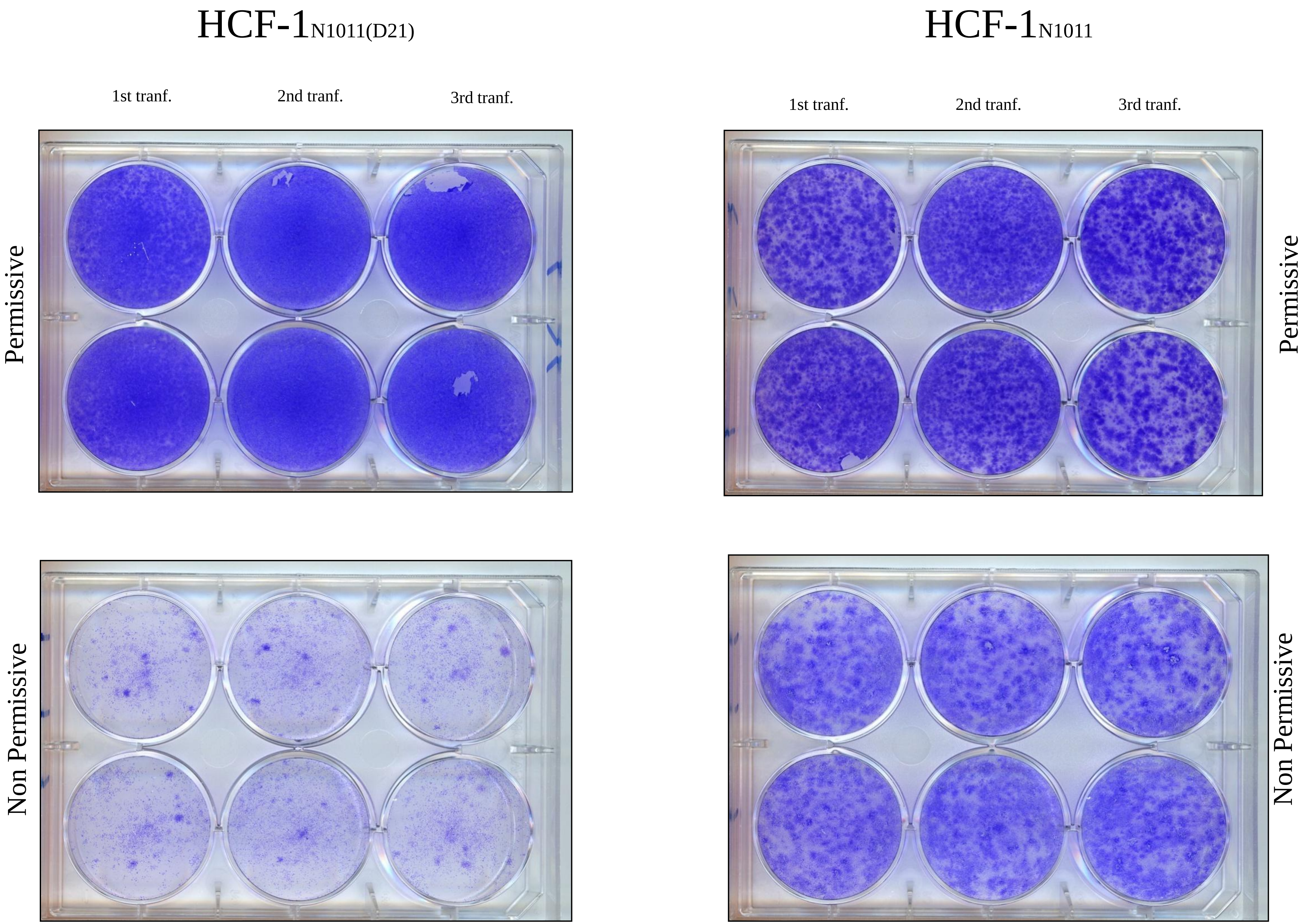

HCF-1N1011(D21)
HCF-1N1011
1st tranf.
2nd tranf.
3rd tranf.
1st tranf.
2nd tranf.
3rd tranf.
Permissive
Permissive
Non Permissive
Non Permissive
