# Supplementary material for: Role of the HCF-1 Basic Region in Sustaining Cell Proliferation
Source: PLoS One. 2010 Feb 2;5(2):e9020. doi: 10.1371/journal.pone.0009020 (PMC2814863; doi:10.1371/journal.pone.0009020)
Supplement: Figure S4 — Stable Expression of selected HCF-1 N-terminal deletion mutants in HeLa cells. (0.36 MB PDF) [file pone.0009020.s005.pdf]

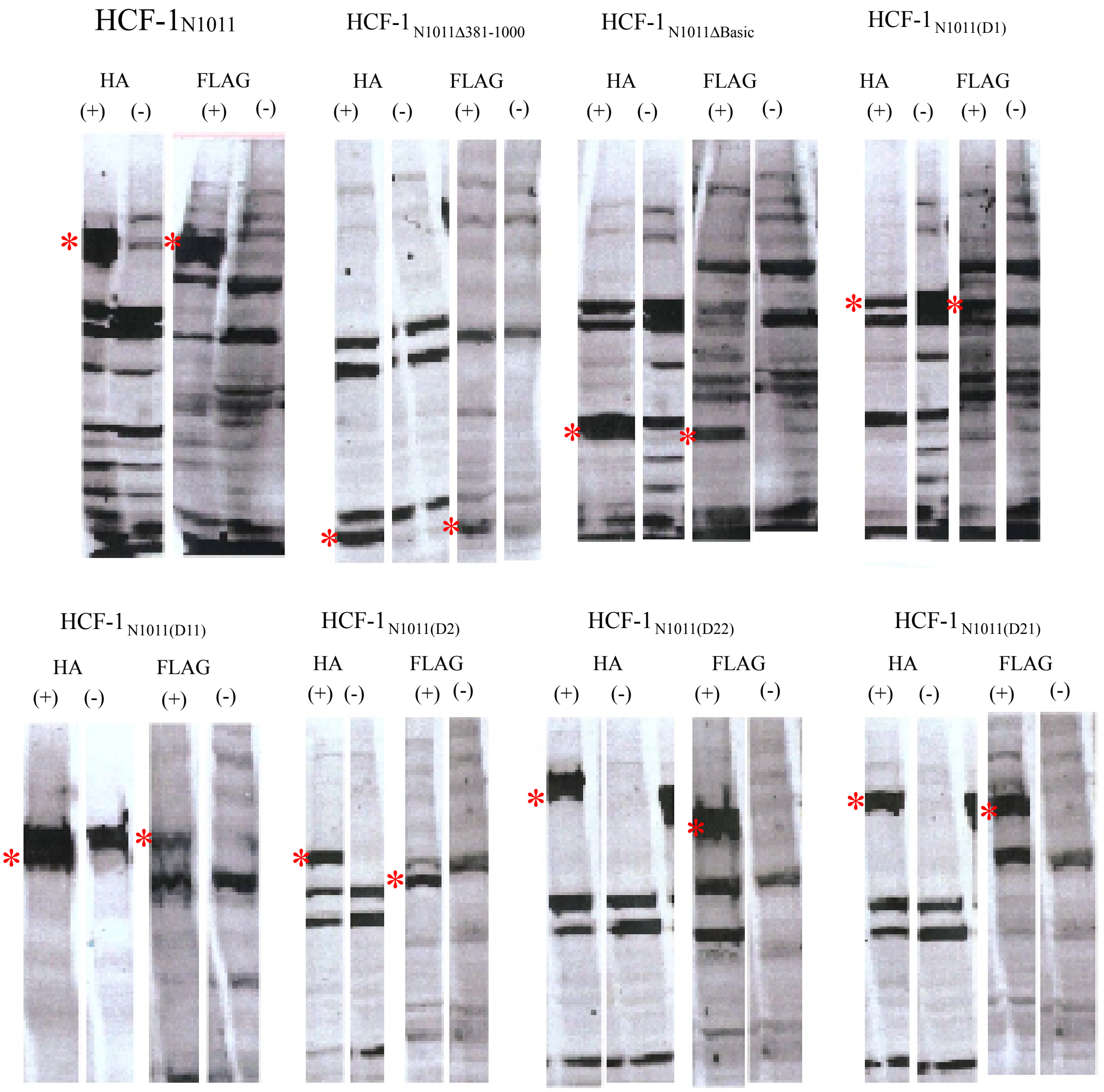

Supplemental Figure 4: Stable Expression of selected HCF-1 N-terminal deletion mutants in HeLa cells. Each mutant has been tested with HA and FLAG antibodies (red asteriscs) and is shown with 293 wt cells (-)
